# Seascape Genomics of the Sugar Kelp *Saccharina latissima* along the North Eastern Atlantic Latitudinal Gradient

**DOI:** 10.3390/genes11121503

**Published:** 2020-12-13

**Authors:** Jaromir Guzinski, Paolo Ruggeri, Marion Ballenghien, Stephane Mauger, Bertrand Jacquemin, Chloe Jollivet, Jerome Coudret, Lucie Jaugeon, Christophe Destombe, Myriam Valero

**Affiliations:** 1 UMI EBEA 3614, Evolutionary Biology and Ecology of Algae, CNRS, Sorbonne Université, UC, UACH, Station Biologique de Roscoff, CS 90074, Place Georges Teissier, 29688 Roscoff CEDEX, France; jaroguzinski@gmail.com (J.G.); P.Ruggeri@nhm.ac.uk (P.R.); marion.ballenghien@sb-roscoff.fr (M.B.); stephane.mauger@sb-roscoff.fr (S.M.); bertrand.jacquemin@ceva.fr (B.J.); jollivet.chloe@gmail.com (C.J.); jerome.coudret@sb-roscoff.fr (J.C.); ljaugeon@sb-roscoff.fr (L.J.); destombe@sb-roscoff.fr (C.D.); 2 Department of Bacteriology, Animal and Plant Health Agency, Addlestone KT15 3NB, Surrey, UK; 3 Xelect ltd, Horizon House, Abbey Walk, St Andrews KY16 9LB, Scotland, UK; 4 UMR 7144, Adaptation et Diversité en Milieu Marin, CNRS, Sorbonne Université, Station Biologique de Roscoff, CS 90074, Place Georges Teissier, 29688 Roscoff CEDEX, France; 5 CEVA, 83 Presqu’île de Pen Lan, 22610 Pleubian, France; 6 Ecole polytechnique de Lausanne (EPFL), SV-IBI UPOATES, Route cantonale, CH-1015 Lausanne, Switzerland

**Keywords:** local adaptation, genetic diversity, outlier loci, environmental variables, phylogeography, SNPs and microsatellites

## Abstract

Temperature is one of the most important range-limiting factors for many seaweeds. Driven by the recent climatic changes, rapid northward shifts of species’ distribution ranges can potentially modify the phylogeographic signature of Last Glacial Maximum. We explored this question in detail in the cold-tolerant kelp species *Saccharina latissima*, using microsatellites and double digest restriction site-associated DNA sequencing ( ddRAD-seq) derived single nucleotide polymorphisms (SNPs) to analyze the genetic diversity and structure in 11 sites spanning the entire European Atlantic latitudinal range of this species. In addition, we checked for statistical correlation between genetic marker allele frequencies and three environmental proxies (sea surface temperature, salinity, and water turbidity). Our findings revealed that genetic diversity was significantly higher for the northernmost locality (Spitsbergen) compared to the southern ones (Northern Iberia), which we discuss in light of the current state of knowledge on phylogeography of *S*. *latissima* and the potential influence of the recent climatic changes on the population structure of this species. Seven SNPs and 12 microsatellite alleles were found to be significantly associated with at least one of the three environmental variables. We speculate on the putative adaptive functions of the genes associated with the outlier markers and the importance of these markers for successful conservation and aquaculture strategies for *S. latissima* in this age of rapid global change.

## 1. Introduction

The extent and distribution of genetic variation within a species are of fundamental importance to its evolutionary potential, and determine its chances of survival. Assessments of genetic variation are therefore essential for the development of effective conservation strategies and for the management of genetic resources [1,2].

The spatial distribution of genetic diversity within and between populations is the result of gene flow, genetic drift, and natural selection. Historical events, like the succession of expansion and contraction in species’ ranges during the glacial period and afterwards during post-glacial recolonization, are thought to be a major factor influencing the genetic diversity patterns currently observed in a wide variety of organisms [3,4]. Such historical and demographic events are thought to be at the origin of a commonly observed latitudinal gradient of genetic diversity, with high regional genetic diversity at the low latitude rear edge and genetic homogeneity at the high latitude leading edge for temperate species [5,6], or higher genetic diversity at the high latitudes for Arctic and cold-tolerant species [7,8,9]. The regional pattern of diversity at the rear edge is characterized by high levels of genetic differentiation among small, disjointly distributed, genetically depauperate populations that had undergone recurrent bottlenecks [5,6]. However, as the distribution of species can be strongly controlled by temperature, such latitudinal gradients of genetic diversity may have also arisen due to selective effects [10]. For example, due to global warming, loss of the most diverse populations has been reported for numerous species [11], in particular for marine kelp forests [12]. In the context of the management of genetic resources, it is crucial to know which populations are more diverse and could therefore serve as a “reservoir” of genetic diversity [13,14].

Elucidating the drivers of the genetic structure of populations observed across the range of a species thus requires a clear understanding of the relative influences of the historical processes resulting from stochastic demographic effects and the selective processes, particularly those linked to global warming. Specific regions of the genome can be affected by adaptive selection processes. Modern approaches in molecular biology allow to characterize this adaptive variation even in non-model organisms, providing tools to answer fundamental and applied questions in evolution and ecology [15,16]. The widespread use of single nucleotide polymorphisms (SNPs) as genetic markers about twenty years ago has greatly increased the number of loci available to researchers thus enabling more precise testing of neutral or stochastic genetic variation models. The higher number of available genetic markers also allows for predictive quantification of adaptive variation, hence informing researchers about deterministic processes such as selection [17]. Despite known statistical biases, utilization of a genome-wide panel of a large number of SNPs can allow for detection of outlier loci that are potentially under selection and/or are linked to genes that are important for local adaptation. These methodologies can enable the discovery of putative associations between SNPs and environmental factors that likely impact genetic structure of the studied species [18,19]. Additionally, there are now increased possibilities for the identification of the function of the candidate genes (see [20]).

Kelp forests are considered as one of the most diverse and productive temperate and cold-water ecosystems [13,21]. For these marine brown algae, temperature is generally regarded as the most important range limiting factor since their reproduction (gametogenesis and sporogenesis) is particularly sensitive to rising temperatures [22,23,24]. In the last few decades, a significant change in the distribution of kelp forests at a global scale has been widely reported (for a review see [25]), and the scientific consensus is presently that global warming is severely threatening these marine ecosystems. Detailed investigations of the patterns of genetic diversity along the European coasts have been conducted mainly for a number of temperate or cold-tolerant marine species. The general trend revealed a reduction in genetic diversity with increasing latitude that was linked with episodes of contraction/expansion of the species' ranges during the Last Glacial Maximum (LGM) [6,26,27]. Recently, [28] challenged the existing paradigm that Arctic marine kelp forests are a depauperate extension of temperate populations and instead emphasized the persistence of Arctic refugia through the cycles of glaciation. Levels of connectivity between marine forests of a number of European kelp species have been investigated at different spatial scales ranging from less than 1 km to in excess of hundreds of kilometers (*Laminaria digitata*: [29,30,31,32]; *L. hyperborea*: [29,33,34]; *L. ochroleuca*: [14]; *Saccorhiza polyschides*: [27]; *Saccharina latissima*: [34,35,36,37,38]). All of these studies agreed that the levels of connectivity were generally very low. This implied that the negative consequences of the threats brought about by climate change cannot be mitigated by population connectivity, which makes the scenario of extirpation of a large number of populations and hence widespread loss of genetic diversity highly likely. In this context, populations located in the central part of a species’ distribution range (i.e., the more genetically diverse populations) are generally considered to be less vulnerable to the predicted warming trends compared to the marginal populations (that may already be genetically depauperate) (*L. digitata*: [32,39]; *Sargassum fallax* and *Scytothalia dorycarpa*: [40]). The extent of local adaptation depends on a complex balance between selection, drift, and gene flow. Selection is more effective in large, well-connected populations because drift is reduced, but on the other hand gene flow, by reintroducing "poorly adapted genes", can restrict local adaptation. However, even in small populations, the effect of genetic drift can be counterbalanced in a highly selective marginal environment, and local adaptation processes might be favored [32,39].

*Saccharina latissima* (Linnaeus) (sugar kelp) is a brown macroalga (Phaeophyceae) that belongs to the family Laminariaceae and has a circumpolar distribution. It is found in cold and temperate coastal waters of the lower intertidal with a lower depth limit of about 30 m [41,42]. In the Arctic, this cold-tolerant species has been reported to complete a full life cycle under perennial ice [43]. *S*. *latissima* forms largely continuous dense stands on rocky shores, occurring in both clear and murky waters, preferably in areas sheltered from strong waves [44]. In Europe, *S*. *latissima*’s range extends from Spitsbergen in the north to the northern part of Portugal in the south. Multiple studies suggested that *S*. *latissima* has a circumpolar origin [14,38,45]. Existence of at least three major *S*. *latissima* ice age refugia (located in Northern Iberia, the Celtic Sea, and Iceland) has been proposed based on ecological niche modelling [14]. Previous phylogeographic studies examined patterns of *S*. *latissima* genetic diversity but their sampling regime either did not account for the entire European range of this species (Iberian Peninsula to the Arctic Ocean) [37,38], or their study did not focus strictly on the European Atlantic coastline [45]. Therefore, the occurrence of the putative Arctic or Iberian Peninsula refugia, and the importance of these refugia to postglacial recolonization of *S*. *latissima* compared to the other proposed refugia currently is not clear. The dispersion and settlement of *S*. *latissima* propagules requires hard rocky substrate coastal environments and its populations cannot establish in places where there are long stretches of sandy coastline [22]. The wide latitudinal and vertical distribution of *S*. *latissima* suggests the occurrence of local adaptation along its distribution range. Indeed, the presence of ecotypic differentiation between populations has been reported with regard to temperature (between two western Atlantic populations, [46]; Arctic and cold-temperate waters populations of the eastern Atlantic: [24,47,48]; salinity [24], and increased pCO_2_ [48]). 

In this study we analyzed the structure and genetic diversity of *S*. *latissima* along the European coast in light of the previously hypothesized refugia areas, their putative role in post-glacial recolonization, and the importance of connectivity. We also conducted *F_ST_* outlier analysis to identify loci potentially associated with several environmental variables at the Pan-European scale. Investigation of population genetic structure was performed using expressed sequence tag (EST)-derived [37] and genomic-derived [49] microsatellite (SSR) loci, and newly developed ddRAD-seq SNP markers. Hence, as we used the next generation sequencing data, our study benefited from an increased amount of detected polymorphisms compared to previous studies in our sampling range of this species.

## 2. Materials & Methods

### 2.1. Sampling, DNA Extraction, and Genotyping

**Figure 1:**
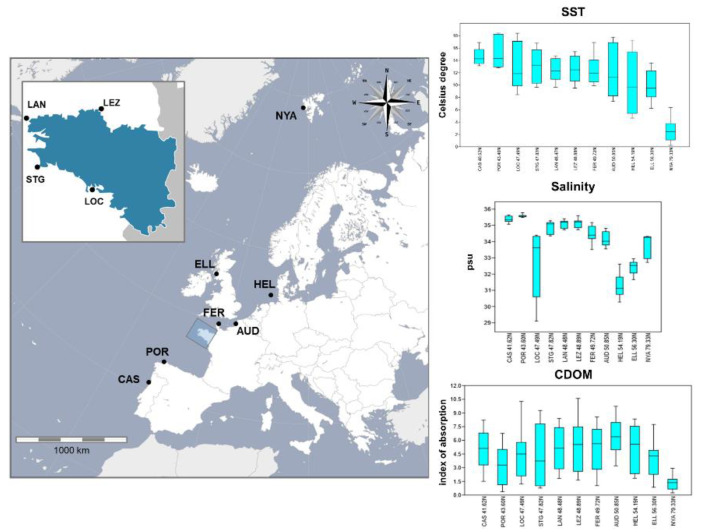
Map of the 11 sampling sites for the *S.* *latissima* sporophytes analyzed in this study. The insert shows the precise position of the four Brittany (France) sampling localities. See Supplementary Materials Table S1A for the detailed description of the 11 sampling sites. The box-plots shown on the right-hand side of the map display within-site variation for three environmental variables: sea surface temperature (SST), salinity, and chromophoric dissolved organic material (CDOM). The localities (x-axis) are ordered by latitude (south to north from left to right).

*S*. *latissima* sporophytes were sampled between 2014 and 2016 at 11 sites situated along a latitudinal transect parallel to the European Atlantic coast. The transect ranged from northern Portugal (latitude 41.622) to Spitsbergen in the Arctic Ocean (latitude 79.334), covering both the putative LGM refugia of this species as well as the post-glacially colonized areas (Figure 1, Supplementary Materials Table S1A). *S*. *latissima* sporophytes were collected in the lower intertidal zone and by diving in the higher subtidal zone (+1 to −5 m depth), except for samples from Ellenabeich (ELL) where the sporophytes were collected as drift material from the beach. As the sporophyte density was low at the sampling sites Castelo do Neiva (CAS) and Fermanville (FER), fewer than 15 individuals were collected from these localities. A small disc of tissue was cut at the base of the blade from each of the sampled individuals (a non-lethal procedure). Dried tissue samples were preserved in individual zip-locked plastic bags containing silica gel and stored at room temperature until DNA extraction.

DNA extraction was performed on between 10 and 20 mg of dried tissue utilizing the Nucleospin^®^ 96 II kit (Macherey-Nagel GmbH & Co. KG, Düren, Germany) following the manufacturer’s protocol. The lysis step was performed at room temperature rather than at 65 °C to minimize extraction of polysaccharides (which may interfere with PCR amplification). 

Double digest restriction site-associated DNA sequencing (ddRAD-seq) library construction and sequencing (the latter carried out at Fasteris, Switzerland) were performed as described in [50]. A larger number of *S*. *latissima* individuals, a subset of which were used in this study, were sequenced in eight libraries, two of which were in part sequenced twice due to insufficient number of reads resulting from the initial sequencing effort. Three individuals were sequenced in each of the ten libraries to assess the consistency of the genotyping.

Read demultiplexing (process_radtags module), and locus assembly and variant calling (denovo_map.pl module), were performed in Stacks 1.40 [51]. The populations module of Stacks was used to retain only those loci that were polymorphic and present in at least 90% of the analyzed samples. The resulting variant call format (VCF) file was imported into R programming language for further filtering. This included removal of individuals for which there was more than 10% of missing data, and, in order to minimize linkage disequilibrium in the dataset, random removal of all but one SNP if multiple SNPs were present within the same ddRAD-seq read. These steps, in addition to selection of samples from localities most relevant for the study of genetic diversity along a latitudinal gradient, led to the retention of 199 individuals from 11 localities (Table 1, Supplementary Materials Table S1A), and 7511 polymorphic SNP markers. These markers were further reduced to 4069 (originating from 4069 loci) after minor allele frequency (MAF) selection performed in the radiator R package [52]. SNPs were retained if the within-locality MAF exceeded 0.04 or if the global MAF exceeded 0.01. In order to account for the fact that several sampling localities comprised fewer than ten individuals (Table 1), SNPs were not retained if they had passed the within-locality MAF threshold only in these low sample number localities. The final dataset exhibited 2.41% missing data which, after imputation of the missing genotypes (utilizing a random forest algorithm in radiator R package), was reduced to 0.14% missing data. For detailed description of the methods on the construction of the ddRAD-seq libraries, ddRAD-seq locus assembly, variant calling, and SNP filtering see Supplementary Materials Text. The conversion to different input formats from the VCF file was done in R or in PGD-Spider [53]. 

SSR locus amplification and scoring of ten EST-derived SSR loci was performed as detailed in [37], and for eight genomic SSR loci as described in [49] (see Supplementary Materials Text for names of the loci used). Alleles were sized using the SM594 size standard [54] and scored manually using the software GeneMapper 4.0 (Applied Biosystems, Foster City, CA, USA). The CAS locality was excluded from the SSR analysis due to very poor PCR amplification. A total of 280 *S*. *latissima* sporophytes collected at ten sites were genotyped with the SSRs (Table 1).

### 2.2. Genetic Diversity and Analysis of Population Structure 

All the analyses described below were performed in the same manner for both the SNP and the SSR marker types unless otherwise stated.

For each of the localities we computed several within-locality genetic diversity indices. Allelic richness (Ar) was obtained for each locus for the SNPs utilizing the allelic.richness function from R package hierfstat [55], whereas for the SSRs Ar was obtained from the allel.rich function of the PopGenReport [56] R package. Both methods performed allelic richness computations with rarefaction. For each locality we report average Ar across all of the genotyped loci. The number of alleles private to each locality was computed, summed across the localities, and converted to a percentage of the total number of private alleles. For the SNPs this was done only for those loci that did not exhibit any missing data at any of the localities for the total number of 2337 private alleles. For the SSRs, there were a total of 37 private alleles. Within each locality, the observed heterozygosity, *H_O_*, the expected heterozygosity, *H_E_*, and the fixation index, *F_IS_*, were computed in the genepop 1.0 [57] R package. *H_E_* was calculated for each locus and subsequently averaged across loci to obtain a within-locality metric. Significance of the departure from the Hardy-Weinberg Equilibrium (HWE) within each locality (*F_IS_*) was computed with an exact test in genepop 1.0 utilizing dememorization = 100,000, batches = 5000, and iterations = 10,000.

In order to estimate how the localities differed from one another in terms of within-locality expected heterozygosity, we performed a locus-by-locus pairwise Welch’s t-test among the examined localities (i.e., for each of the 4069 loci for the SNPs and for 18 SSR loci). Corrections for multiple comparisons were performed using the p.adjust function in R to control for the false discovery rate (FDR) [58]. Furthermore, to investigate the correlation in the variation of the within-locality genetic diversity with latitude, the within-locality Ar, percentage of private alleles, and *H_E_* were plotted against the latitude of each of the localities. The significance of the correlation between these genetic diversity indices and the latitude was tested in R with a Pearson’s product-moment correlation. 

To investigate the genetic structure of the populations, Arlequin 3.5.2.2 [59] was used to compute the pairwise *F_ST_* values amongst all the localities. The significance of the comparisons was tested using 10,000 permutations (an alpha error was set at 5%). The R function p.adjust was employed to control for FDR in order to correct for multiple comparisons. As all of the pairwise *F_ST_* comparisons were high and significant (see Results), the degree of genetic variation between and within the sampling localities was further assessed using discriminant analysis of principal components (DAPC). This analysis, performed in the R adegenet package [60], clusters individuals by refining genetic differentiation between populations while minimizing within-population differences. The a-score criterion was used to decide how many principal components to retain during the DAPC, and hence to avoid overfitting. Each individual was a priori specified as belonging to the site it was sampled from (thus the DAPC for the SNPs was performed on 11 a priori clusters, and for the microsatellites on 10 a priori clusters). DAPC outputs were visualized via scatterplots of the first two or the second and the third principal components and via bar plots displaying the probabilities of assignment of individuals to each of the analyzed localities (the compoplot function of adegenet).

To further explore levels of population structure amongst the sampling localities, a likelihood-based genetic clustering was performed using the snapclust function of adegenet [61]. The optimal number of genetic clusters, k, was determined with the snapclust.choose.k function which was used to identify the optimal k among k values ranging from one to twice the number of sampled localities. The optimal k was selected based on the Akaike information criterion (AIC) output of snapclust.choose.k for each k (the lower the AIC, the better the fit), visual inspection of the k versus AIC chart, and the outputs of other analyses. Subsequently the assignment of sporophytes to each of the optimal k clusters was computed and explored by running the snapclust algorithm at the selected k, with the pop.ini argument specified as ward (the initial groups were defined using the Ward algorithm), and max.iter = 10,000. The probability of membership of each individual to each of the k clusters was visualized using compoplot. 

Isolation-by-distance (IBD) for both marker types was assessed by plotting the geographical distance (the shortest distance between localities along the coastline in km) against the genetic distance (*F_ST_*/(1- *F_ST_*)), and computing the significance of the IBD using Mantel test in Genetix 4.05 [62] with 1000 permutations.

### 2.3. Description of the Environmental Variables

Environmental variable data were extracted from the SeaDataNet (http://gher-diva.phys.ulg.ac.be/web-vis/) and ERDDAP (https://coastwatch.pfeg.noaa.gov/erddap/griddap/erdMHcdom8day.html) web-servers (Figure 1, Supplementary Materials Table S1A) for each of the studied localities (Figure 1). The environmental variables comprised i) sea surface temperature (SST), ii) salinity, and iii) chromophoric dissolved organic material (CDOM). All of these environmental features are directly related to, or can represent a proxy for, important physiological responses in *S*. *latissima*. SST and salinity are parameters that can define limits of tolerance for the maintenance of stable *S*. *latissima* populations over time [63], whereas CDOM represents the density of organic material suspended in the water column and is an excellent proxy for i) bacterial abundance, ii) turbidity related to organic material resuspension, and iii) PAR (photosynthetic active radiation) [64]. The SST, salinity, and CDOM data were obtained as follows: SST (degrees Celsius) was retrieved from the SeaDataNet server as monthly measurements (January to December) averaged over the 1900–2014 timeframe. Similarly, sea surface salinity (psu) was obtained for each month of the year as a monthly average over the 1900–2013 timeframe. CDOM (absorbance at 412 nm) data were extracted from the MODIS ERDDAP database as measurements collected for August over the 2002–2013 timeframe. The values for each of these three environmental variables are summarized for each of the 11 *S*. *latissima* sampling localities in Supplementary Materials Table S1A. For each environmental variable we computed four environmental parameters: 1—the average, 2—the minimum value, 3—the maximum value, and 4—the coefficient of variation (cvar) (standard deviation/average), an independent estimation of the variation of an environmental variable in each locality. The cvar for SST and salinity was estimated from averaging the monthly measurements (that refer to the mean values per month over a centennial scale); cvar-SST and cvar-salinity refer therefore to the intra-annual variation within each locality, i.e., variation in SST and salinity among the different months in a single year. The cvar-CDOM was estimated from averaging in each locality the CDOM values collected each year in the 2002–2013 timeframe; therefore, the cvar-CDOM refers to variation in CDOM across different years.

### 2.4. Test for Detection of Candidate Outlier Loci

SNP and SSR markers were screened for candidate outliers using the Arlequin 3.5.2.2 [59] fdist function [65]. This method for the detection of candidate outlier loci is based on the examination of the joint distribution between *F*_ST_ and *H*_E_. The relation between *F*_ST_ and *H*_E_ can lead to two different scenarios of selection: directional selection (when there is an uncoupling between *F*_ST_ and *H*_E_ with loci showing larger genetic differentiation for the corresponding *H*_E_ values) or balancing selection (when the genetic differentiations is below the threshold of expectation for the estimated *H*_E_ values).

The fdist method offered the possibility to detect all the possible candidate outliers based on comparisons among a total of 55 pairwise tests for the SNPs (pairwise tests conducted amongst 11 localities) and 45 pairwise tests for the SSRs (pairwise tests conducted amongst 10 localities). This method, in turn, provided a compressive list of loci that exhibited non-neutral behavior due to both the *S*. *latissima* life history and/or the current local selective processes. This methodology, based on correlation between allele frequencies and environmental data variation, was preferred to other environmental correlation methods (i.e., Bayenv [66]) because i) not all of the environmental parameters considered in this study exhibited a gradient of variation with latitude [67,68], and ii) because it enabled direct comparison of the candidate outliers for the two marker types used in this study (as the fdist method can be applied to both SNPs and SSRs). We further utilized the hierarchical island method (HIM) in Arlequin to distinguish outliers created by demography from those putatively under selection. HIM was performed separately for each marker type, with the hierarchical population structure specified a priori based on the genetic clusters derived from the snapclust algorithm described above. To limit the possibility of type 1 error (discovery of false positives), a 5% FDR was applied to the list of the candidate outlier loci for each marker type.

The potential function of a gene in which each putative outlier marker was located was investigated using a nucleotide-BLAST search against Genbank (https://blast.ncbi.nlm.nih.gov/Blast.cgi). The ddRAD-seq consensus sequence tag (150 bases) of each candidate outlier SNP was used to perform a nucleotide-BLAST search using the default alignment settings. This procedure replicated a methodology previously performed in similar studies by other authors [69]. Only alignments that displayed an E-value < 0.001 (number of expected hits equivalent to those found by chance) were considered significant. The list of the candidate outlier loci with significant BLAST matches was annotated and only those markers linked to genes were retained.

### 2.5. Environment-Genome Multivariate Correlation Tests

The SNP outlier loci that were putatively associated with a gene were analyzed for a statistical correlation with 12 environmental parameters (four different measures: 1—the average, 2—the minimum value, 3—the maximum value, and 4—the coefficient of variation for each of the three environmental variables: SST, salinity, and CDOM).

An autocorrelation test was performed in PAST 3.26 [70] in order to define the level of redundancy among the three environmental variables. Each of the 12 environmental parameters was independently linearly regressed against the allele frequencies of the candidate SNP and SSR outliers. For the SSRs, alleles with frequencies below 5% across the entire dataset were removed from the test. Multiple regression tests were performed in PAST 3.26 and a 5% FDR correction was applied to the list of significant loci. In order to visualize the relationship between the environmental and genomic variation, a set of heat-map graphs that correlated allele frequencies with variation in the 12 environmental parameters was produced with the online software heatmapper (http://heatmapper.ca/) [71].

## 3. Results

### 3.1. ddRAD-seq Genotyping Effort and Locus and SNP Marker Filtering

Sequencing of ddRAD-seq libraries yielded a total of 1,294,743,101 reads (on average 2,380,042 reads per sample), which, after initial filtering steps, were assembled into 1,377,662 loci. Locus filtering resulted in 13,122 polymorphic loci and 103,963 SNPs remaining. This set of SNP markers was further reduced to 4069 SNPs that were utilized in the study.

### 3.2. Genetic Diversity

Several within-locality genetic diversity measures were computed for multiple *S*. *latissima* sampling sites situated along a latitudinal gradient spanning the entire European Atlantic range of this species (Figure 1). Both SNP and SSR markers were used to obtain the within-locality genetic diversity (Table 1). The results show a high degree of congruence between the two types of marker used in this study in terms of the within-population genetic diversity patterns across the sampled range (Table 1). The *H_E_* values were relatively low for both the SNPs and the SSRs for the CAS and Portiño de Dexo (POR) populations at the southern limit of the range of *S*. *latissima*. The *H_E_* values were higher, and equivalent to one another, within the four French Brittany sites (Locmariaquer (LOC), St. Guenolé (STG), Lanildut (LAN), Lézardrieux (LEZ)). These sites and the surrounding coastline are characterized by the presence of long stretches of continuous rocky shores. There was a marked decrease in *H_E_* for the populations sampled from the eastern part of the English Channel and beyond (FER to Helgoland (HEL)). All these sites are situated on rocky shores but are separated from each other by large areas of sandy substrate. This is especially the case for the Helgoland archipelago (HEL), which exhibited the lowest *H_E_* of all sites for the two types of markers (Table 1). The two northernmost sites displayed the highest within-population heterozygosity of all the sampling sites. The within-locality *H_E_* values were 5 to 13 times higher for the SSRs than for the SNPs (Table 1).

The observed genetic diversity patterns were supported statistically by the outputs of the pairwise Welch’s t-test that compared *H*_E_ for all pairs of populations, and especially so for the SNPs (Table S2A). Values of within-population heterozygosity were significantly higher for the northernmost site (Ny-Ålesund (NYA)) compared to the two southernmost sites (CAS and POR) (Supplementary Materials Table S2A). The SSR results (Supplementary Materials Table S2B) indicated that all sites had similar *H*_E_, although the comparisons involving HEL with the two northernmost sites were significant before the FDR correction. This difference in results between SNPs and SSRs possibly stems from the fact that the SNP comparisons were based on a much higher number of loci. 

For SNPs, the highest genetic diversity was again found for the northernmost population (NYA) when measured in terms of the percentage of private alleles whereas the lowest value was again found for HEL (Table 1). This pattern was not exactly the same for the SSRs. For the other sites, the variation was not concordant between the two marker types (Table 1). This could be partly explained by the fact that (1) SNPs are bi-allelic compared to the multi-allelic SSRs, and (2) the number of within-population private alleles is dependent on sample size. Allelic richness globally showed a very similar pattern of variation with latitude to *H*_E_ for both marker types, with much reduced diversity within Audresselles (AUD) and especially within HEL (Table 1). However, the highest values were not observed for the northernmost site NYA, but for ELL. 

Taken together, the results presented above indicate that there was no significant variation in genetic diversity with latitude across the 11 study sites. The Pearson's product-moment correlation of *H*_E_, allelic richness, and percentage of private alleles with latitude was always non-significant for both marker types (Supplementary Materials Table S3).

### 3.3. Deviation from Random Mating

The within-locality *F*_IS_ values were broadly congruent between the two marker types, ranging between −0.130 and 0.145 for the SNPs and between −0.062 and 0.125 for the SSRs (Table 1). Several localities exhibited significant departures from HWE for the SNPs: LEZ, AUD, and NYA (significant heterozygote deficiency), and POR and HEL (significant excess of heterozygotes). None of the localities had significant departures from HWE for the SSRs, but the general trends of the SSR within-locality *F*_IS_ values closely matched those observed for the SNPs (Table 1). 

### 3.4. Population Structure 

Similar patterns of genetic differentiation were observed for the two marker types even if the absolute pairwise *F*_ST_ values were generally higher for the SNPs than for SSRs (Supplementary Materials Table S4). All populations were significantly genetically differentiated, with the lowest pairwise *F*_ST_ found intra-Brittany between the close-by STG and LAN locations (0.060 for the SNPs and 0.036 for the SSRs). The highest *F*_ST_ values were observed between HEL and the southernmost or the northernmost localities (*F*_ST_ greater than 0.700 for the SNPs and greater than 0.400 for the SSRs, Supplementary Materials Table S4), revealing strong genetic isolation of the Helgoland archipelago (HEL). The same pattern, but to a lesser extent, was observed for the AUD sampling site, located on a rocky spot at the entrance to the North Sea. Nevertheless, a significant signal of isolation by distance was detected in the whole dataset for both marker types (Supplementary Materials Figure S1A for the SNPs, Supplementary Materials Figure S1B for the SSRs), though the signal was stronger when the locality of Spitsbergen (NYA) was removed from the isolation by distance analysis (i.e., for the SSRs Mantel statistic: G = 2.570, *p*-value < 0.001, (Pearson coefficient *r* = 0.724); without NYA, G = 3.008, *p*-value < 0.01, (*r* =0.869); and for the SNPs: G = 2.639, *p*-value < 0.01, (*r* = 0.633); without NYA, G = 3.904, *p*-value < 0.001, (*r* = 0.873)).

**Figure 2:**
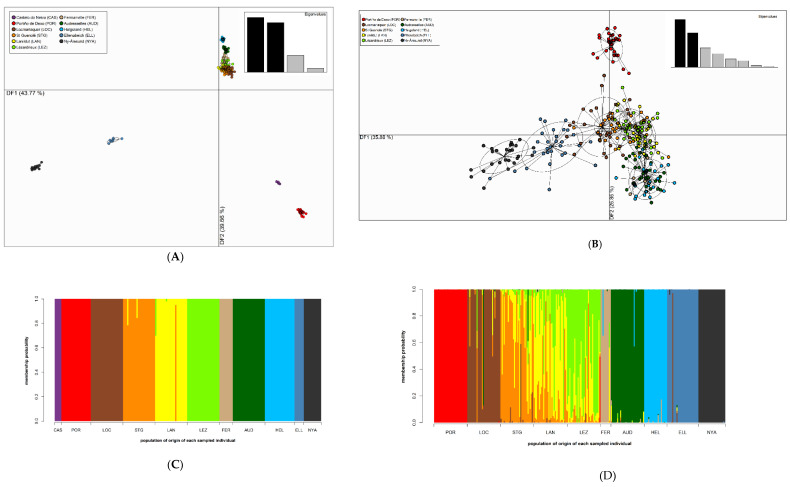
Discriminant analysis of principal components (DAPC) scatterplot and compoplot for (**A**,**C**) 199 *S*. *latissima* sporophytes sampled from 11 localities (see key) that were genotyped at 4069 SNP markers, and (**B**,**D**) 280 *S*. *latissima* sporophytes sampled from 10 localities (see key) genotyped at 18 SSR markers. (**A**,**C**) display the first two components (axes 1 and 2) of the DAPC. Sampling localities, which were specified a priori for the DAPC, are differentiated by color and inertia ellipses. Each point corresponds to a single individual. (**B**,**D**) show DAPC posterior probability of membership of each of the analyzed *S*. *latissima* sporophytes (each sample is represented by a vertical bar) to each of the sampling sites (each represented by a unique color).

Clustering of the localities by DAPC was highly congruent with the patterns of pairwise genetic differentiation amongst the sampling sites. This was the case for both marker types, which exhibited highly similar DAPC clustering patterns although with more clear subdivision of the sampled populations when based on the SNPs (Figure 2, Supplementary Materials Figure S2). For the SNPs, the two first axes of the DAPC explained more than 80% of the variability (Figure 2A), and axes two and three close to 45% of the variability (Supplementary Materials Figure S2A). In accordance with the first axis, the populations followed a more or less latitudinal gradient: the southernmost and the northernmost populations were clustered at the two extremes whereas there was a tight cluster of the Brittany sites in the middle (relatively low pairwise genetic differentiation), and close by were the clusters representing the eastern English Channel and the North Sea entrance sampling sites (Figure 2A). The populations located at the southern or northern edges of the *S*. *latissima* distribution were again clearly separated on the second axis from the more central sites (Supplementary Materials Figure S2A). To a lesser extent, axis two further separated the FER, AUD, and HEL localities but noticeably the four Brittany sites still clustered together (Supplementary Materials Figure S2A). For the SSRs (Figure 2B and Supplementary Materials Figure S2B, for the two first axes—which explained over 60% of variability, and for axes two and three—which explained nearly 40% of variability, respectively), these patterns were less clear but there was again a marked separation of the southernmost and northernmost sites from one another and from the more central sampling localities. These patterns were further underlined by the compoplots displaying the membership probability of each sporophyte to each of the sampled localities (Figure 2C,D). The levels of admixture were greatest between the neighboring localities, particularly within Brittany, and much more prevalent for the SSRs (Figure 2D) than for the SNPs (Figure 2C).

**Figure 3:**
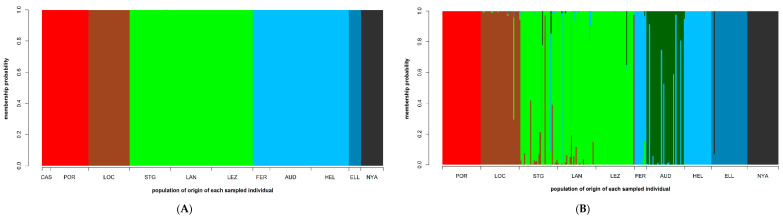
(A) Membership probability of the 199 S. latissima sporophytes (each sample is represented by a vertical bar) to each of six genetic clusters (each represented by a unique color) that were determined by the snapclust algorithm that was run on this set of samples utilizing 4069 SNP markers (see Supplementary Materials Figure S3A,B). The sampling locality of each individual is specified along the x-axis. (B) Membership probability of the 280 S. latissima sporophytes (each sample is represented by a vertical bar) to each of seven genetic clusters (each represented by a unique color) that were determined by the snapclust algorithm that was run on this set of samples utilizing 18 SSR markers (see Supplementary Materials Figure S3C,D). The sampling locality of each individual is specified along the x-axis.

The most parsimonious number of snapclust clusters according to the AIC criterion (but also to other considerations such as other analyses presented herein and knowledge of the biogeography of the studied sites) was six for the SNPs (Supplementary Materials Figure S3A) and seven for the SSRs (Supplementary Materials Figure S3C). The assignment of the populations to these genetic clusters were similar between the marker types but the resolution was better for the SNPs compared to the SSRs (Figure 3A and Supplementary Materials Figure S3B for the SNP markers, and Figure 3B and Supplementary Materials Figure S3D for the SSR markers). The six genetic clusters defined by the SNPs grouped the localities according to their geography: a southernmost cluster comprising populations from the Iberian Peninsula (CAS and POR); a southern Brittany cluster composed of only one population (LOC); a north-western Brittany cluster (STG to LEZ); a cluster grouping localities from the eastern section of the English Channel up to the entrance to the North Sea (FER to HEL); and the two northernmost clusters, each made up of a single locality (ELL and NYA) (Figure 3A, Supplementary Materials Figure S3B). These six genetic clusters were also retrieved for the SSRs (Figure 3B, Supplementary Materials Figure S3D), but in addition a seventh cluster was observed that separated out the population at the entrance of the North Sea (AUD). This cluster was admixed with the cluster that grouped the localities that were closest geographically (FER and HEL) (Figure 3B, Supplementary Materials Figure S3D). The snapclust clustering was not totally congruent with the DAPC. For instance, for the SNP markers for the DAPC there was no clear separation of individuals from the four Brittany sites (Figure 2A), whereas according to snapclust, LOC clearly forms its own separate cluster (Figure 3A). This could partly be explained by the fact that LOC is the most highly genetically differentiated locality according to the pairwise *F*_ST_ values (SNP markers) amongst the four Brittany localities (Supplementary Materials Table S4).

Population genetic structure assessed after removal of putative outlier SNPs (3730 loci remaining) and SSRs (13 loci remaining), see below, was broadly congruent with the results obtained with full marker sets. See Supplementary Materials Text and Figures S4–S7 for details.

### 3.5. Test for Detection of Candidate Outlier Loci and Correlations with the Environmental Parameters

**Figure 4:**
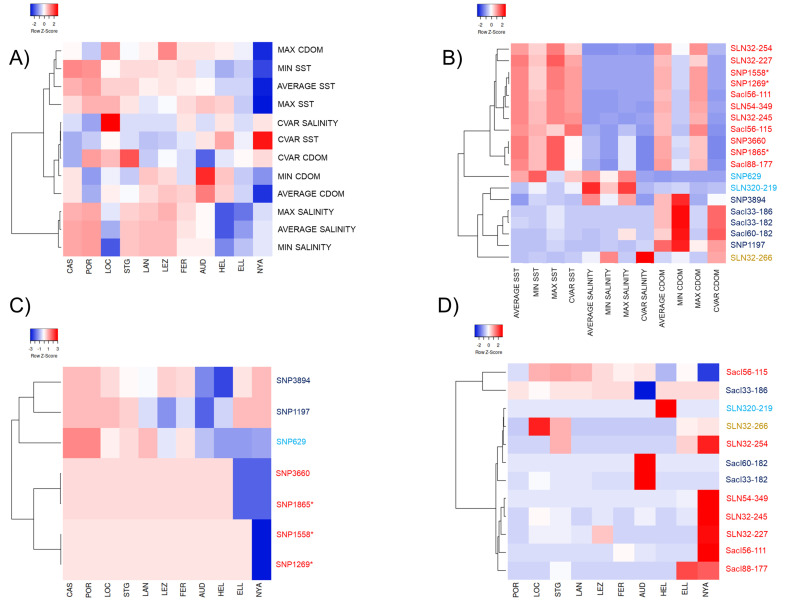
Heat-map graphs with co-association dendrograms for *S*. *latissima*. (**A**) Pairwise Spearman’s ⍴ correlations between 12 environmental parameters plotted according to their geographical variation. (**B**) Variation in the coefficient of determination (R^2^) resulting from the correlations between 19 markers (seven SNPs and 12 microsatellite alleles) and 12 environmental parameters. (**C**,**D**) Geographical variation in allele frequencies of (**C**) SNP, and (**D**) SSR markers shown on (**B**). The localities (*x*-axis for (**A**,**C**,**D**)) are ordered by latitude (a south to north gradient). In panels (**B**–**D**), SNP and SSR locus labels are highlighted in different colors to represent four main clusters of associations of the loci with 12 environmental parameters. Loci marked with * represent markers for which the allele frequency patterns were more likely influenced by *S*. *latissima* demography than by the selective processes (see explanation in the Material and Methods section “Test for detection of candidate outlier loci").

The test for assessing correlation amongst the 12 parameters associated with the three environmental variables (SST, salinity, CDOM) showed six significant outcomes out of 66 possible pairwise tests. These six tests showed significant autocorrelation between parameters belonging to SST and salinity but not between any parameters belonging to the different environmental variables. For SST, average SST correlated significantly with all the other SST parameters (avg-SST vs. min-SST, *r* = 0.946, *p* < 0.001; avg-SST vs. max-SST, *r* = 0.898, *p* < 0.05, and avg-SST vs. cvar-SST, *r* = −0.911, *p* < 0.01; min-SST vs. cvar-SST, *r* = −0.930, *p* < 0.01) (Figure 4A, Supplementary Materials Table S1B). For salinity, average salinity correlated significantly with both min-salinity (*r* = 0.922, *p* < 0.01) and max-salinity (*r* = 0.962, *p* < 0.001) (Figure 4A, Supplementary Materials Table S1B).

The fdist screening for candidate outliers detected a total of 339 candidate outlier SNPs (out of 4069 loci), and 10 SSR loci that potentially deviated from neutrality (five that were derived from the genomic library: SLN32, SLN35, SLN54, SLN319, SLN320, and five EST-derived loci: Sacl33; Sacl56; Sacl60, Sacl78, Sacl88). Out of the 339 candidate outlier SNPs, 40 significantly matched (E-value < 0.001) to sequences in Genbank. These outliers represented 11.80% of the examined candidate outliers (40/339), and 0.98% of the total SNP set (40/4069). The 40 candidate outliers matched 97 different sequences originating from 23 different taxa that belonged to seven different phyla of five kingdoms (see Supplementary Materials Figure S8). The largest proportion of the 97 matches was to brown algae derived sequences (58.76%) (Supplementary Materials Figure S8). Further, 26 out of 40 candidate outliers showed significant association with functional genomics traits.

The search for candidate outliers that imposed a hierarchical island method (HIM) identified 7 out of 26 candidate outlier SNPs that might reflect the historical population structure of *S*. *latissima* (Table 2A). It also highlighted one SSR locus (SLN35) out of the 18 examined that was significantly associated with the effects of hierarchical structure (Table 2A). 

Multiple correlations of the outlier locus allele frequencies with the 12 environmental parameters showed significant correlations at seven SNP loci, involving 10 of the 12 environmental variables (average, min, max, and cvar-SST; average and max salinity; average, min, max, and cvar-CDOM) (Table 2B). In detail, five SNP loci (SNP629, SNP1269, SNP1558, SNP1865, SNP3660) correlated significantly (*p*< 0.05) with the SST parameters (including average, min, max, and cvar-SST); one SNP locus (SNP629) correlated significantly with salinity (including average and max salinity), and five SNP loci (SNP1197, SNP1269, SNP1558, SNP1865, SNP3894) correlated significantly with CDOM (including average, min, max, and cvar-CDOM) (Figure 4B, Table 2B).

Multiple correlations performed on the outlier SSR markers revealed a total of 12 alleles belonging to seven different loci that correlated significantly with a set of ten environmental variables (average, min, max, and cvar-SST; average, max, and cvar-salinity; average, min, and max-CDOM) (Supplementary Materials Table S5). Furthermore, seven SSR alleles (Sacl56-111, Sacl56-115, Sacl88-177, SLN32-227, SLN32-245, SLN32-254, SLN54-349) correlated significantly (*p* < 0.05) with the SST parameters (including average, min, max, and cvar-SST); two SSR alleles (SLN32-266, SLN320-219) correlated with salinity (including average, max, and cvar-salinity); and eight SSR alleles (Sacl33-182, Sacl33-186, Sacl56-111, Sacl56-115, Sacl60-182, SLN32-245, SLN32-254, SLN54-349) correlated significantly with CDOM (including average, min, and max-CDOM) (Figure 4B, Supplementary Materials Table S5).

The graphical projection of the coefficient of determination (*R*^2^) for the SNPs and SSR alleles which correlated significantly with the 12 environmental parameters identified four main clusters of associations. The first cluster comprised four SNPs (SNP1269, SNP1558, SNP1865, SNP3660) and seven SSR alleles (Sacl56-111, Sacl56-115, Sacl88-177, SLN32-227, SLN32-245, SLN32-254, SLN54-349) (Figure 4B) that showed a general pattern of positive correlation with all SST and average/max-CDOM parameters and a negative correlation with salinity and cvar-CDOM (see the loci marked in red on Figure 4B). A second cluster comprised two SNPs (SNP1197, SNP3894) and three SSR alleles (Sacl33-182, Sacl33-186, Sacl60-182) showing positive correlation with average, min-CDOM, and cvar-CDOM (see loci in dark blue on Figure 4B). A third cluster comprised one SNP (SNP629) and one SSR allele (SLN320-219) that correlated positively with average and max-salinity (see loci in light blue on Figure 4B). The fourth cluster was represented by a single SSR allele (SLN32-266) that correlated positively with both min-salinity and cvar-salinity (see locus in ochre on Figure 4B).

The correlation of allele frequency variation within the outlier SNP loci and environmental parameters identified three main clusters of loci with respect to how these loci behaved within the different sampling localities (Figure 4C). The first cluster suggested joint variation of allele frequencies at four loci (SNP1269, SNP1558, SNP1865, SNP3660). These loci were positively associated with SST and CDOM and major allele frequencies seemed to be negatively correlated with these environmental features in the northernmost localities like ELL and NYA (Figure 4C, red text). The second cluster was represented by loci SNP1197 and SNP3894, both positively associated with average and min-CDOM. These loci exhibited the lowest major allele frequency within the Channel Sea (LEZ, AUD) and the North Sea (HEL) localities (Figure 4C, dark blue text). The third cluster comprised just the locus SNP629, positively associated with all SST parameters except max-SST and average and max-salinity. For this locus, major allele frequency positively correlated with SST in the southernmost localities (CAS and POR) and negatively correlated with SST in the northernmost localities (AUD, HEL, ELL, NYA) (Figure 4C, light blue text).

Concerning the three EST-derived microsatellite loci (Sacl33, Sacl56, and Sacl60) that were significantly correlated with the environmental parameters, the association patterns revealed pairs of alleles belonging to the same locus for which each allele showed opposite patterns of correlation with the same environmental parameter. Specifically, alleles Sacl33-182 and Sacl33-186 were both correlated with min-CDOM but with positive and negative correlation, respectively (Figure 4D, Supplementary Materials Table S5). The allele Sacl60-182 was also positively correlated with min-CDOM and displayed an allele frequency pattern that was similar to the allele frequency pattern of the allele Sacl33-182 (Figure 4D, Supplementary Materials Table S5). Furthermore, alleles Sacl56-111 and Sacl56-115 showed opposite correlation patterns with both the average-SST and cvar-SST (both negative for Sacl56-111 and both positive for Sacl56-115) (Figure 4D, Supplementary Materials Table S5). No linkage disequilibrium was observed among any of these loci in both SNPs and SSRs, therefore suggesting independence of genetic variation observed at these loci.

## 4. Discussion

### 4.1. Patterns of Variation in Within-Locality Genetic Diversity

In this study we investigated the genetic diversity and population structure of cold-tolerant kelp *S*. *latissima* sampled along a latitudinal gradient accounting for the entire eastern Atlantic distribution range of this species. Our results demonstrated a high degree of inter-population genetic differentiation with an absence of significant variation in genetic diversity with latitude. There are several potential non-mutually exclusive reasons, including both historical and contemporary causes, which may explain the reported patterns of *S*. *latissima* genetic diversity. The location of the ice-free refugia a species was occupying during the LGM has been proposed to have had a major influence on the genetic diversity patterns observed across that species’ present-day range [3]. For European marine species, ice-free LGM refugia are thought to have been located mainly in the regions of Iceland/Faroe Islands, the Celtic Sea, and the Iberian Peninsula [26]. In our study, the greatest genetic diversity was found in the northernmost localities of NYA (Arctic Ocean) and ELL (Scotland), which partly confirms the patterns of genetic diversity previously observed for European *S*. *latissima* [37,38,45]. However, our observation of the highest levels of heterozygosity and the percentage of private alleles for the northernmost Arctic Ocean population gives strong support to the niche modelling prediction of an *S*. *latissima* Arctic LGM refugia proposed by [14]. This result also supports the statement of [28] proposing to “revise our understanding of Arctic marine forests and how they persist through cycles of glaciation”. The absence of any gradient of variation of genetic diversity with latitude could potentially be explained by the occurrence of two other putative LGM refugial areas of *S*. *latissima*, as suggested by [14], one located in the Celtic Sea (here Brittany, LAN) and the other in northern Iberia (POR). Our results indicate that the populations sampled in these regions exhibited high levels of the percentage of private alleles. It is possible that the Iberian Peninsula ice-free refugia played a lesser role in the post-LGM expansion of *S*. *latissima* compared to the refugia located further north, depending on whether there was long-term persistence of mostly continuous distribution of this species from Iberia to the English Channel, or if alternatively there was a lack of gene flow between the two distinct refugial zones. Given that we observed the highest levels of genetic diversity within the two northernmost localities of our sampling range, it might also be that the high latitude *S*. *latissima* populations presently inhabiting the formerly glaciated regions originated exclusively from the refugia that were closest to them (i.e., the Arctic Ocean LGM refugia), resulting in genetically divergent populations currently found at the lower latitudes. Thus, most likely the southern refugia did not substantially contribute to the post-glacial recolonization of the high latitude areas.

On the other hand, the relatively low genetic diversity of the Iberian *S*. *latissima* populations could potentially be explained by the rising seawater temperatures at the southern edge of distribution of this temperate-cold adapted seaweed species. Such a change in the environmental conditions could be highly detrimental to *S*. *latissima* [25,72], resulting in small fragmented populations at the southernmost edge of its eastern Atlantic distribution range, leading to low effective population size, diminished inter-population gene flow, low within-locality genetic diversity, and ultimately extinction of populations located in this low latitude rear edge area. Based on recent records of density fluctuations, such a scenario was validated for the populations of kelp *Saccorhiza polyschides* occupying the rear edge of this species’ distribution range in southern Portugal [27]. However, for *S*. *polyschides*, persistence of high genetic diversity at the regional level within the southern Portugal rear edge area occurred despite the drastic population size reductions. For *S*. *latissima*, it may be that the recent extinction of the majority of populations in the Iberian Peninsula (northern Portugal/Galicia) rear edge area driven by climate change has erased the more ancient phylogeographic signature of genetic diversity.

There was a broad concordance of the patterns of *S*. *latissima* within-population genetic diversity and population genetic structure deduced based on the two marker types employed in this study. Although the within-locality expected heterozygosity values that we had obtained were 5 to 13 times higher for the SSRs than for the SNPs depending on the analyzed population, importantly the relative differences in *H_E_* between the studied populations were highly similar based on both marker types. Similar variations in the magnitude of within-locality *H_E_* between SSRs and SNPs have been observed before [73] and are likely due to methodological and technical differences rather than due to biological differences. In comparison to the SSRs, SNP markers afforded higher resolutive power for describing fine-scale population genetic structure. Such overall concordance between SNPs and SSRs, but taking into account the difference in resolutive power between these two types of marker, has been reported in other recent studies of marine and freshwater organisms, including kelp [50], brown trout [74], coral reef-associated fish [75], and pike [73]. The SNP derived *H_E_* values that we observed were approximately an order of magnitude lower than those obtained for several western Atlantic *S*. *latissima* localities [76]. Whether this difference is due to contrasting demographic histories or different responses of the *S*. *latissima* populations on either side of the Atlantic Ocean to environmental change or rather from the different methodologies used to obtain the SNP markers in our study and in [76] is something that should be investigated further.

### 4.2. Low Connectivity Levels between Disjunct S. latissima Sampling Sites

Overall, the strong genetic structuring that we report here for *S*. *latissima* is in agreement with previous analyses of the population structure in this kelp species [33,37,76]. Compared with the previous published studies, the high discriminant power of in excess of 4000 SNP markers developed as part of this study suggests that these markers could prove very useful for kelp aquaculture to detect individual migrants (or genetic pollution) between eastern Atlantic *S*. *latissima* populations when separated by at least 300 km. The high and significant pairwise *F_ST_* values revealed that there was substantial genetic differentiation between all pairs of the populations we analyzed, whereas the DAPC suggested that the sampling localities were largely independent entities, as there were only a few occurrences of individuals that were not assigned to their locality of origin or that displayed mixed ancestry, and in those instances the exchange of migrants largely took place between the nearby localities. In addition, even though the snapclust algorithm grouped the sampled localities into fewer genetic clusters than there were sampling sites, each cluster was clearly composed of only neighboring sampling sites, thus suggesting that long range exchanges of migrants are an unlikely occurrence. Nevertheless, the occurrence of well separated genetic clusters for each of the southernmost populations is in agreement with what is expected at the rear edge of the distribution of *S*. *latissima* and provides support for the existence of the Iberian Peninsula *S*. *latissima* LGM refugia. The identification of the AUD locality as an independent cluster by the snapclust analysis performed with the SSR markers contrasted with the output of snapclust algorithm run with the SNPs. This apparent discrepancy could be explained by the hypothesis that the SSR locus Sacl-33 was under selection at this locality, as demonstrated by its significant statistical association with the CDOM environmental parameter . Indeed, when this locus was removed and the snapclust analysis was rerun on a neutral SSR marker panel, the AUD locality was included in the same genetic cluster as the neighboring English Channel localities of FER and HEL. The above results were further supported by a significant IBD signal for both marker types, even with the NYA locality included in the analyses. The substantial geographic distance separating NYA and the other sampling localities meant that there likely was very little gene flow between NYA and the other sampling sites, and thus not in agreement with the stepping-stone IBD hypothesis.

The extremely low within-population genetic diversity of the AUD and HEL localities deserves a special mention. It suggests that these populations experienced elevated levels of genetic drift that can be explained by their geographic isolation (“island” of rocky substrate surrounded by several hundreds of kilometers of sandy beaches) and their low effective population size. We predict that these populations will become extinct in the near future. Such low levels of genetic diversity within the HEL locality was also reported for the kelp *L*. *digitata* [39], and maladaptation of that population to the local conditions was thus suggested. However, contrary to what was expected if drift was the major driver of evolution, *L*. *digitata* samples from the Helgoland archipelago exhibited the weakest heat stress response in [39]. This result suggested that in this isolated, genetically depauperate, population local adaptation still appeared to be taking place, potentially because strong selective forces towards the upper thermal limit of *L*. *digitata* might have counterbalanced the deleterious effects of genetic drift. Examination of non-neutral genetic variation could therefore provide a more complete understanding of the potential for local adaptation [15,16,77,78].

### 4.3. Adaptive Functional Responses to the Seascape in S. latissima

Oceans are multifaceted and dynamic environments in both space and time where the patchy vertical (depth) and horizontal (geographical) distribution of various environmental stressors/features is likely to be an important driver of the local adaptive responses of all marine organisms, a phenomenon that can be better understood utilizing “seascape genetics” (reviewed in [79,80,81]). “Seascape genetics” aims to uncover signs of local adaptive responses at both historical and recent time scales. This is of particular relevance considering that there is increasing evidence that the physical and biochemical changes in the seascape due to global warming are threatening the long-term persistence of many kelp forests, especially at the marginal areas of their distribution [24,82]. Here, we examined how geographical variation in three major abiotic parameters (temperature, salinity, and light) might interact with the genetic diversity of S. *latissima* and assessed how this may have influenced historical and recent local adaptive processes. In our study, we identified seven outlier SNPs putatively involved in functional adaptive responses. Three SNPs (SNP1269, SNP1558, SNP1865) showed allele frequency patterns that have likely been driven by historical events that shaped *S*. *latissima* population structure during the cycles of expansion and contraction of the species range following the LGM [38,45]. These three SNPs appear to be related to SST and CDOM parameters and were found to be potentially part of genomic sequences coding for i) the tic20 protein, ii) the heat shock protein 70 (hsp70), and iii) a female-specific marker Msj68/58/2.

The tic20 protein plays a role in the transport of proteins into the chloroplast in plants and ensures the correct functioning of photosynthesis [83,84]. This gene, which has been found in Phaeophyceae, has been predicted as a candidate quantitative trait locus (QTL) marker that controls growth of blade in *Saccharina japonica* [85]. Not much is known about how the tic20 gene responds to temperature and light stress in macroalgae but a significant decrease in transcript abundance of this gene was observed in *Pisum sativum* after exposure to higher temperatures [86]. Similarly, the hsp70 gene has been largely demonstrated to be directly involved in physiological processes that renature proteins damaged by thermal stress in a wide range of organisms, including kelp [87,88]. It is, therefore, logical to presume that both the tic20 and the hsp70 genes were involved in important adaptive processes that led *S*. *latissima*, a typical cold-adapted species, to expand and establish permanent populations in the warmer environments that characterize its current range of distribution.

Furthermore, we observed a significant link between the *S*. *latissima* SNP1865 marker and the female-specific marker Msj68/58/2 from *S*. *japonica* [89]. Although sex markers having been identified in other brown algae [90], no female-specific markers have yet been characterized for *S*. *latissima*, suggesting that an investigation of the SNP1865 genomic sequence could help to uncover this aspect. The allelic variation at SNP1865 was significantly correlated with variation in SST and CDOM, suggesting the existence of an evolutionary sex-specific effect driven by these two environmental features. The idea that SST and light stressors might cause evolutionary differences at the sexual haploid stages (gametophytes) of *S*. *latissima* has been already suggested by several authors [91,92,93]. A recent comparative analysis of the transcriptome of *S*. *latissima* male and female gametophytes revealed that temperature has important implications for physiological processes which produced gender-specific effects in *S*. *latissima* such that the male gametophytes were able to tolerate higher temperatures than the female gametophytes [94]. This evidence might reinforce the potential adaptive role of SNP1865 and its linkage with a putative *S*. *latissima* female-specific marker considering that the divergent north-south allele frequency patterns at the SNP1865 marker strongly suggest a role for temperature along the examined latitudinal gradient. However, further investigations should be carried out to validate the correlation between temperature and the putative *S*. *latissima* female-specific marker SNP1865, as it has recently been suggested that genomic areas exposed to higher rates of recombination (e.g., several regions of sex chromosomes) might lead to false positive divergence of the detected outliers in genome scan analyses [78,95].

Among the remaining four outlier SNPs that correlated with SST, CDOM, and salinity, we identified potential associations with genes involved in alginate (phosphomannose isomerase 1 gene ,c5epi gene, and mannuronan C-5 epimerase gene) and iodine (vanadium-dependent iodine peroxidase gene) metabolic pathways. These SNPs might be involved in local selective processes that may enhance the survival of sporophytes that experience high levels of variation in SST, CDOM, and salinity. Phosphomannose isomerase 1 (PMI1) (SNP629 and SNP1197) and the mannuronan C-5 epimerase (MC5Es) (SNP3660) are key genes in the metabolic pathways that lead to the biosynthesis of mannitol and alginate carbohydrates [96]. Alginates are the main components of the cell wall in kelp, while mannitol represents the major carbon storage substance in brown algae [97]. These carbohydrates are important for maintaining kelp homeostasis, especially in stressful environments. Alginates are thought to play an important defensive role in *Saccharina* and might help to resist abiotic stress (due to SST and high salinity) [98,99] and pathogens (also directly correlated with CDOM) [99,100]. Furthermore, increased content of mannitol in adult *S*. *latissima* sporophytes was suggested to be linked to higher seasonal temperature [101]. This hypothesis might be supported by findings in another kelp species, *L*. *digitata*, for which heat-wave experiments implicated SST in varying mannitol content of sporophytes collected from a large latitudinal gradient (low mannitol in Brittany, high in northern Norway) [39]. Vanadium-dependent iodine peroxidase is involved in the biosynthesis of organohalogens (particularly the accumulation of iodine by sporophytes) in all kelp species. Organohalogen accumulation in kelp was suggested as an antioxidative response to reduce the stress resulting from direct exposure to ultraviolet (UV) light at low tide, when the algae are out of the water [102]. A recent transcriptome study showed that vanadium-dependent iodoperoxidase is overexpressed in *S*. *japonica* during events of hyposalinity and hyperthermal stress [103], but not following exposure to direct sunlight. However, the pattern of allele frequencies at the locus SNP3894 (found to be associated with vanadium-dependent iodoperoxidase gene) makes it difficult to speculate on the local selective effects of this gene in *S*. *latissima* in response to CDOM.

Finally, we also observed a high proportion (38.88%) of SSR loci exhibiting non-neutral behavior, half of which were EST-derived markers [37]. EST-SSRs have also been developed for *S*. *japonica* [104] and *L*. *digitata* [105], with some loci shown to be transferable between these two species [105]. The transferability of any of these EST-SSR markers to *S*. *latissima* has not been established [37,104,105]. In this study we detected at least four EST-SSRs (Sacl-33, Sacl-56, Sacl-60, Sacl-88) whose allele frequencies correlated with SST and CDOM environmental parameters (Supplementary Materials Table S5). This result is in agreement with the idea that these EST-SSR loci are linked to genes involved in adaptation to SST and light stress. In fact, the EST-SSR loci described by [37] were developed using the transcriptome from the study of [106] where the authors specifically targeted *S*. *latissima* proteins that responded to acclimation to temperature and light stress.

Among the EST-SSR loci that were correlated with SST and CDOM, loci Sacl56 and Sacl33 stand out. These two loci showed divergent allele frequency patterns (for example, at the localities where the allele Salc56-111 was in high frequency, the allele Sacl56-115 was in low frequency) that might suggest diversifying selection acting on these loci. The nature of the EST-derived SSR loci should allow the genetic variation at these loci to be linked with specific functional traits but we were unable to map any of the analysed EST-SSR markers onto the *S*. *latissima* genome. Generally, diversifying selection can result in significantly high locus-specific differentiation among populations relative to the neutral predictions and the usage of EST-SSRs is perfectly suited to uncover the ecological processes that may be involved in local adaptation [107]. There are not many examples of EST-SSRs associated with diversifying selection in brown algae, but these genetic markers have been widely employed in plants to discover signatures of diversifying selection driven by abiotic ecological features (i.e., in several species of the genus *Eucalyptus*, [108,109]; the tree *Castanopsis fargesii*, [110]; rice *Oryza sativa*, [111]). Future characterization of the Sacl33 and Sacl56 EST-SSRs in relation to the thermal and light stresses might help to predict the performance of different *S*. *latissima* strains under specific aquaculture conditions.

We are aware that the genome-wide scan methods for the identification of outlier loci, here treated as candidate markers associated with adaptive traits, are still too coarse to fully describe the short and long-term evolutionary responses to environmental changes in *S*. *latissima*. There are multiple other possible explanations for the associations between the potential outliers and the environmental responses that we detected in this study. These include the possible existence of (i) strong statistical bias (i.e., false positives), and (ii) the genetic linkage of the identified outlier loci with other distant genes (occurring in low recombinant regions of a chromosome) in the *S*. *latissima* genome. Lacking a fully annotated *S*. *latissima* reference genome, we nonetheless propose that the genome-wide scan approach that we adopted provided an opportunity to predict a set of markers that might suggest physiological adaptive responses. A confirmation for the involvement of these loci in adaptive processes must be validated with future studies designed specifically for this purpose.

In conclusion, our study confirmed the importance of historical demographic processes in shaping the current genetic structure of *S*. *latissima* populations distributed along the European Atlantic coast while also highlighting global warming and the associated environmental changes as an increasingly important factor in driving the distribution range of this kelp species. We observed the highest levels of genetic diversity within the Arctic population, a result which gives support to the hypothesis of the important contribution of the populations that persisted throughout the cycles of glaciation in the LGM Arctic refugia to the present-day patterns of genetic diversity of this species. Six distinct genetic clusters were delimited, knowledge of which will be helpful for the conservation management of the eastern Atlantic populations of *S*. *latissima* as it will allow specific geographic areas containing putatively locally adapted strains to be defined more accurately which is vital for sustainable management of these wild resources for kelp aquaculture. In addition to the recommendation of avoiding the transfer of algae material between disparate genetic clusters and across vast geographic regions because of the potential risks of genetic pollution and/or introduction of foreign pathogens [33,76,112], in the context of spatial and temporal environmental variability, management practices and breeding strategies should also carefully consider the choice of source populations and the selection of varieties suitable for sustainable aquaculture [113]. In this context, the genome-wide outlier scan approach identified several outlier loci that might be involved in differential adaptive responses to the local environmental parameters across our sampling range. However, additional experimental approaches are needed to further confirm the putative selective roles of these genomic regions and their importance for local adaptation, and if they could be used to select potential strains of interest.

## Figures and Tables

**Table 1:** Within-locality genetic diversity indices for 11 *Saccharina latissima* localities genotyped with single nucleotide polymorphisms (SNPs) and 10 localities genotyped with genomic and ESTderived microsatellites (SSRs). N—number of genotyped samples, *H_O_*—observed heterozygosity, *H_E_*—expected heterozygosity, *F*_IS_—inbreeding coefficient (significant values (*p*-value < 0.05) marked with *), Ar—allelic richness (for the SNPs the alleles were rarefied down to ten alleles and for the SSRs the alleles were rarefied down to 44 alleles), and Pr Allele %—percentage of the total number of private alleles (total of 2337 private alleles for SNPs, and 37 private alleles for SSRs). SE—standard error. NA—the analyses were not performed. CAS—Castelo do Neiva, POR—Portiño de Dexo, LOC—Locmariaquer, STG—St. Guenolé, LAN—Lanildut, LEZ—Lézardrieux, FER—Fermanville, AUD—Audresselles, HEL—Helgoland, ELL—Ellenabeich, NYA—Ny-Ålesund.

Population Code	N SNP	*H_O_* (SE) SNP	*H_E_* (SE) SNP	*F*_IS_ SNP	Ar (SE) SNP	Pr Allele % SNP	N SSR	*H_O_* (SE) SSR	*H_E_* (SE) SSR	*F*_IS_ SSR	Ar (SE) SSR	Pr Allele % SSR
CAS	5	0.037 (0.002)	0.034 (0.002)	−0.077	1.086 (0.004)	8.3	NA	NA	NA	NA	NA	NA
POR	22	0.038 (0.002)	0.036 (0.002)	−0.049 *	1.098 (0.004)	12.5	32	0.223 (0.069)	0.228 (0.071)	0.022	2.313 (0.412)	16.2
LOC	24	0.047 (0.002)	0.051 (0.002)	0.074	1.156 (0.005)	13.8	32	0.221 (0.075)	0.231 (0.079)	0.047	2.518 (0.535)	2.7
STG	24	0.050 (0.002)	0.054 (0.002)	0.076	1.173 (0.005)	10.3	32	0.316 (0.080)	0.316 (0.080)	−0.001	3.218 (0.700)	5.4
LAN	24	0.051 (0.002)	0.052 (0.002)	0.022	1.162 (0.005)	5.2	32	0.297 (0.083)	0.289 (0.081)	−0.026	3.230 (0.696)	29.7
LEZ	24	0.046 (0.002)	0.051 (0.002)	0.095 *	1.149 (0.005)	5.1	32	0.305 (0.072)	0.330 (0.077)	0.077	3.128 (0.633)	0
FER	10	0.044 (0.002)	0.045 (0.002)	0.019	1.131 (0.005)	8.8	10	0.211 (0.066)	0.216 (0.068)	0.023	2.228 (0.442)	2.7
AUD	24	0.027 (0.002)	0.027 (0.002)	0.004 *	1.074 (0.004)	2.6	32	0.250 (0.067)	0.249 (0.066)	−0.004	2.328 (0.440)	8.1
HEL	22	0.016 (0.001)	0.014 (0.001)	−0.130 *	1.036 (0.003)	0.6	22	0.192 (0.053)	0.181 (0.051)	−0.062	1.812 (0.247)	8.1
ELL	7	0.055 (0.002)	0.064 (0.002)	0.145	1.177 (0.006)	11.6	30	0.334 (0.068)	0.382 (0.078)	0.125	3.250 (0.626)	8.1
NYA	13	0.056 (0.002)	0.060 (0.002)	0.059 *	1.165 (0.005)	21.1	26	0.340 (0.064)	0.367 (0.066)	0.074	2.775 (0.429)	18.9

**Table:** (**A**)

Locus/Allele	Species Associated with Genbank Hits	Functional Trait/Genome Location	NCBI Accession
SNP1269 (SNP)	*Saccharina japonica*	tic20-like protein gene	KY411551.1
			KY411556.1
			KY411554.1
SNP1558 (SNP)	*Saccharina japonica*	heat shock protein 70 (hsp70) gene	JF507714.1
SNP1691 (SNP)	*Saccharina japonica*	c5epi gene for mannuronan C-5 epimerase	LC053765.1
SNP1865 (SNP)	*Saccharina japonica*	female-specific marker Msj68/58/2 genomic sequence	MF850255.1
SNP2361 (SNP)	*Saccharina japonica*	carbonic anhydrase (CA) gene	KY041784.1
SNP3030 (SNP)	*Chlamydotis macqueenii, Clupea harengus, Picoides pubescens*	solute carrier family 2, facilitated glucose transporter member 1	XM_009907977.1
			XM_012835870.2
			XM_010120537.1
SNP3705 (SNP)	*Saccharina japonica*	heat shock protein 70 (hsp70) gene	JF507714.1
SLN35 (Microsatellite)	*Saccharina latissima*	genomic microsatellite locus	KT723018

**Table:** (**B**)

Locus/Allele	Species Associated with Genbank Hits	Functional Trait/Genome Location	Environmental Correlations	NCBI Accession
SNP629 (SNP)	*Saccharina japonica*	phosphomannose isomerase 1 (PMI1) gene	Min-SST (+) (*R*^2^ = 0.76)	KF937207.1
			Average-Salinity (+) (*R*^2^ = 0.57)	
			Max-Salinity (+) (*R*^2^ = 0.64)	
SNP1197 (SNP)	*Saccharina japonica*	phosphomannose isomerase 1 (PMI1) gene	Min-CDOM (−) (*R*^2^ = 0.66)	KF937207.1
			cvar-CDOM (+) (*R*^2^ = 0.38)	
SNP1269 (SNP) *	*Saccharina japonica*	tic20-like protein gene	Average-SST (+) (*R*^2^ = 0.77)	KY411551.1
			Min-SST (+) (*R*^2^ = 0.53)	
			Max-SST (+) (*R*^2^ = 0.81)	
			cvar-SST (−) (*R*^2^ = 0.70)	
			Average-CDOM (+) (*R*^2^ = 0.64)	
			Max-CDOM (+) (*R*^2^ = 0.71)	
SNP1558 (SNP) *	*Saccharina japonica*	heat shock protein 70 (hsp70) gene	Average-SST (+) (*R*^2^ = 0.77)	JF507714.1
			Min-SST (+) (*R*^2^ = 0.53)	
			Max-SST (+) (*R*^2^ = 0.81)	
			cvar-SST (−) (*R*^2^ = 0.70)	
			Average-CDOM (+) (*R*^2^ = 0.64)	
			Max-CDOM (+) (*R*^2^ = 0.71)	
SNP1865 (SNP) *	*Saccharina japonica*	female-specific marker Msj68/58/2 genomic sequence	Average-SST (+) (*R*^2^ = 0.61)	MF850255.1
			Max-SST (+) (*R*^2^ = 0.68)	
			Average-CDOM (+) (*R*^2^ = 0.51)	
SNP3660 (SNP)	*Saccharina japonica*	c5epi gene for mannuronan C-5 epimerase	Average-SST (+) (*R*^2^ = 0.61)	LC053765.1
			Max-SST (+) (*R*^2^ = 0.68)	
SNP3894 (SNP)	*Saccharina japonica*	vanadium-dependent iodine peroxidase gene	Min-CDOM (−) (*R*^2^ = 0.47)	MG195955.1

Average-SST = average sea surface temperature (in degrees Celsius); Min-SST = minimum sea surface temperature detected in a single year (in degrees Celsius); Max-SST = maximum sea surface temperature detected in a single year (in degrees Celsius); cvar-SST = coefficient of variation of sea surface temperature detected in a single year; Average-Salinity = average salinity (in psu); Min-Salinity = minimum salinity detected in a single year (in psu); Max-Salinity = maximum salinity detected in a single year (in psu); cvar-Salinity = coefficient of variation of salinity; Average-CDOM = average chromophoric dissolved organic material (absorbance at 412 nm); Min-CDOM = minimum chromophoric dissolved organic material (absorbance at 412 nm); Max-CDOM = maximum chromophoric dissolved organic material (absorbance at 412 nm); cvar-CDOM = coefficient of variation of chromophoric dissolved organic material in a single year. (+) = positive correlation; (−) = negative correlation. *R*^2^ = coefficient of determination.

## Data Availability

SNP and microsatellite datasets are deposited in Dryad: https://doi.org/10.5061/dryad.1jwstqjt8.

## Supplementary Materials

The following are available online at https://www.mdpi.com/2073-4425/11/12/1503/s1, Supplementary Text: description of laboratory methods for ddRAD-seq genotyping, description of bioinformatics methods for filtering of ddRAD-seq derived SNPs, and description of population structure results obtained with neutral SNP and SSR marker sets. Figure S1: Mantel test for SNP and SSR markers. Figure S2: DAPC scatterplots for axes 2 and 3 for SNP and SSR markers. Figure S3: outputs of snapclust function for SNP and SSR markers. Figure S4: DAPC scatterplots and compoplot for neutral SNP markers. Figure S5: DAPC scatterplots and compoplot for neutral SSR markers. Figure S6: outputs of snapclust function for neutral SNP markers. Figure S7: outputs of snapclust function for neutral SSR markers. Figure S8: proportion of significant nucleotide-BLAST hits of ddRADseq tags to species, phyla, and kingdoms. Table S1: description of the sampling localities, values of environmental parameters within each sampling locality, and pairwise correlation among the different environmental parameters. Table S2: pairwise comparisons of within-locality *H_E_* for SNPs and SSRs. Table S3: correlation of genetic diversity metrics with latitude for SNPs and SSRs. Table S4: pairwise *F_ST_* for SNPs and SSRs. Table S5: candidate outlier loci SSR alleles that correlated significantly with environmental parameters.
