# Supplementary material for: Seascape Genomics of the Sugar Kelp Saccharina latissima along the North Eastern Atlantic Latitudinal Gradient
Source: Genes (Basel). 2020 Dec 13;11(12):1503. doi: 10.3390/genes11121503 (PMC7763533; doi:10.3390/genes11121503)

**Figure S1** Genetic distance (*F_ST_*/ (1- *F_ST_*)) plotted against the geographic distance (the shortest distance between localities along the coastline in km) for A) 4,069 SNP markers and 11 sampling localities (Mantel statistic G=2.639, p-value<0.01, (Pearson coefficient r=0.633); without Svalbard (NYA), G=3.904, p-value <0.001, (r=0.873)), and B) 18 SSR markers and 10 sampling localities (G=2.570, p-value<0.001, (r= 0.724); without NYA, G=3.008, p-value< 0.01, (r=0.869)).

**A)**
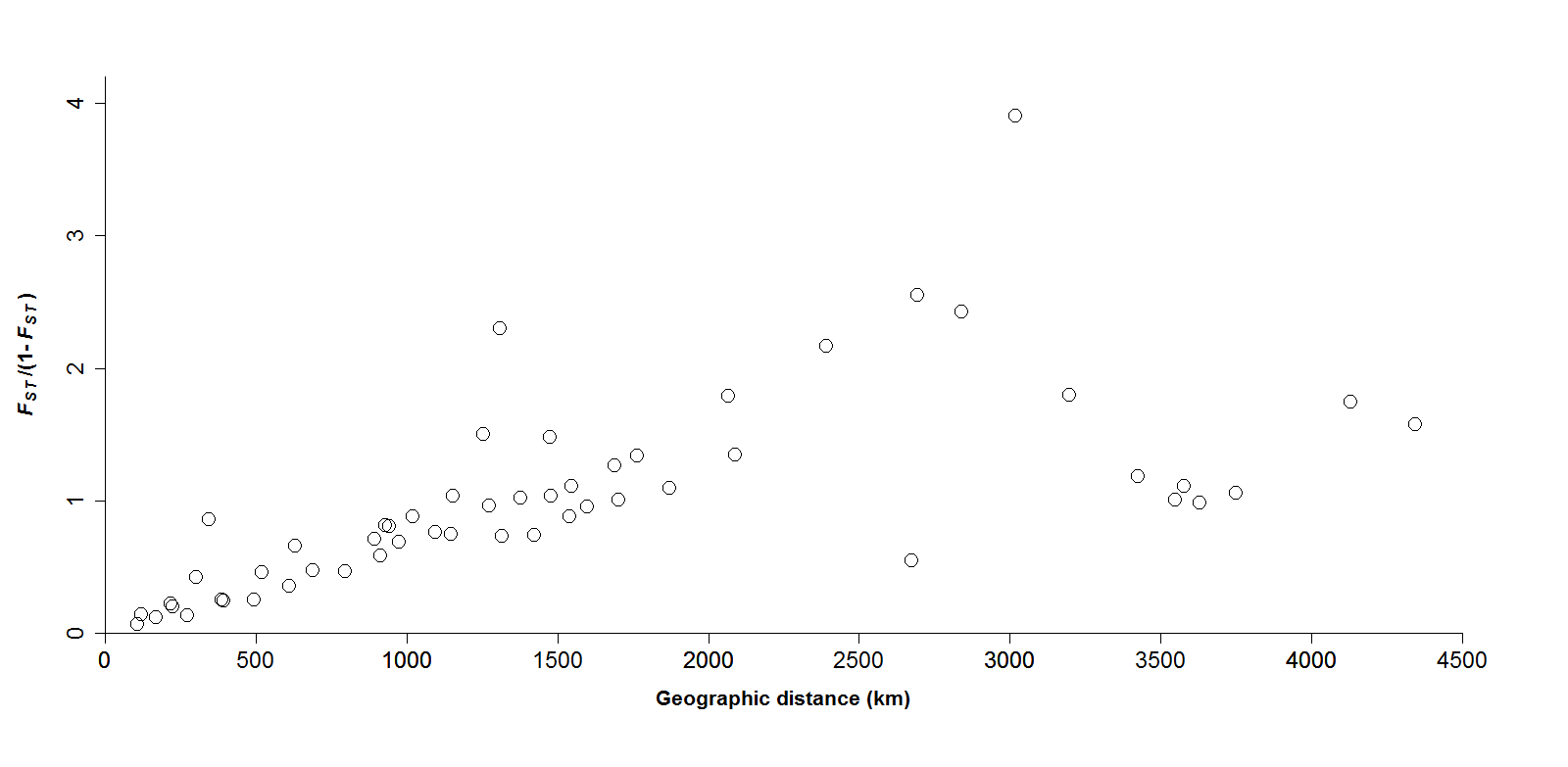


**B)**
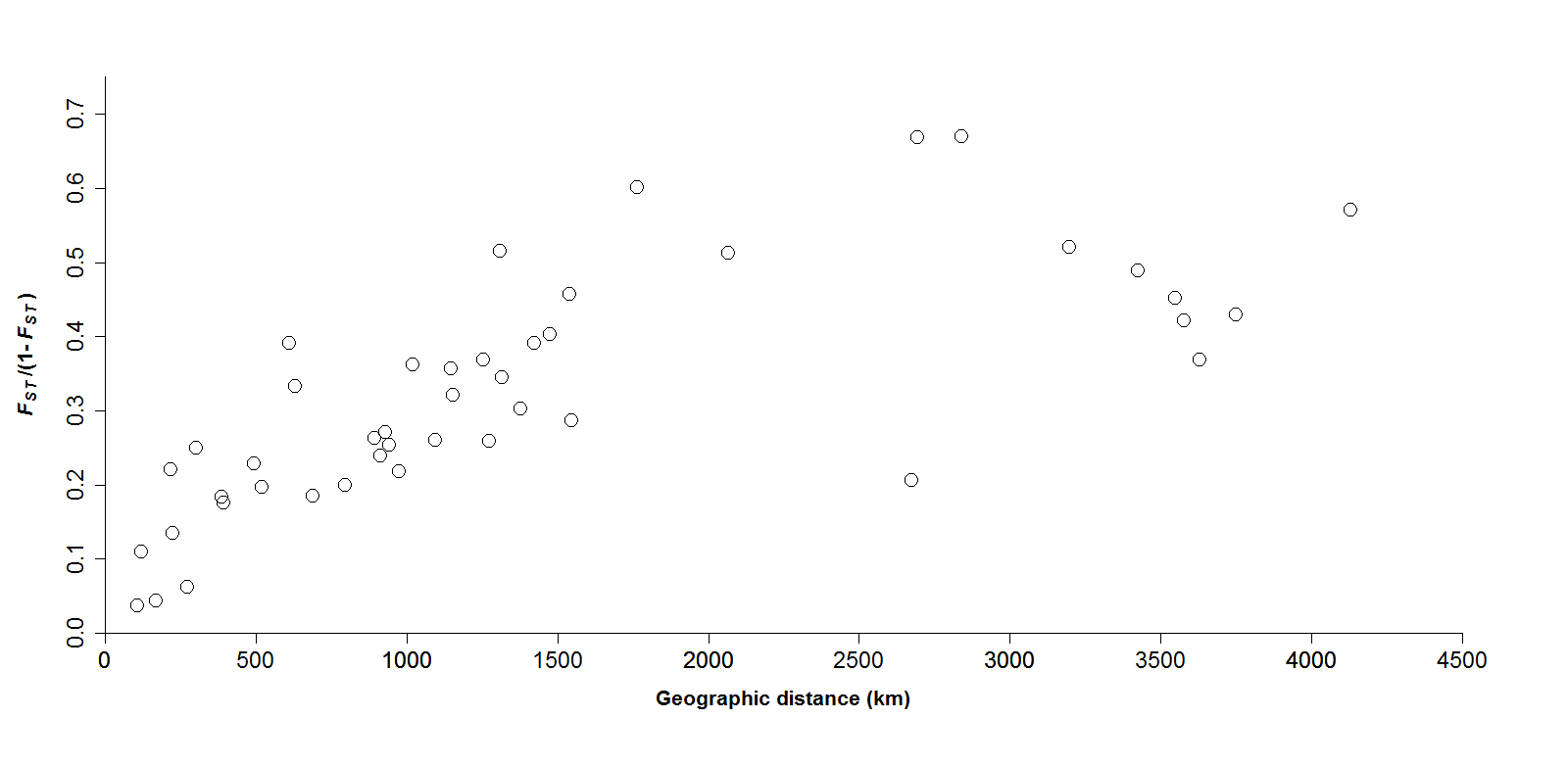


**Figure S2** DAPC scatterplot for A) 199 *S*. *latissima* sporophytes sampled from 11 localities (see key) that were genotyped at 4,069 SNP markers, and B) 280 *S*. *latissima* sporophytes sampled from 10 localities (see key) genotyped at 18 SSR markers. A) and B) display the second and third components (axes 2 and 3) of the discriminant analysis of principal components (DAPC). Sampling localities, which were specified *a priori* for the DAPC, are differentiated by colour and inertia ellipses. Each point corresponds to a single individual.

**A)** **B)**


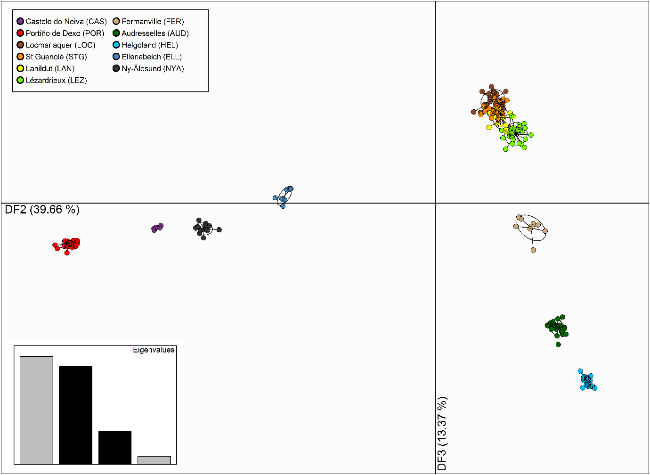

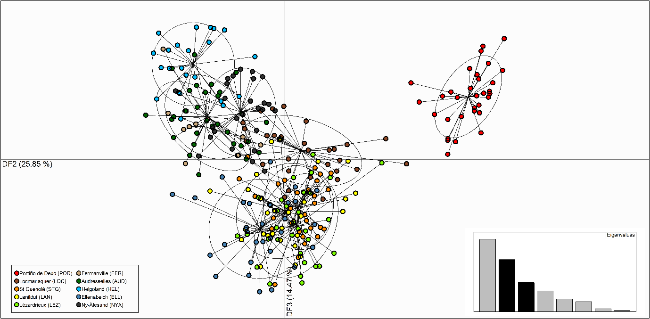


**Figure S3** Output of the *snapclust.choose.k* function of *adegenet* for A) and B) 4,069 SNP markers, and C) and D) 18 SSR markers that was run to determine the optimal number of genetic clusters (*k*) that best describe the genetic structure of A) and B) 199 *S*. *latissima* sporophytes from 11 localities, and C) and D) 280 *S*. *latissima* sporophytes from 10 localities. On A) and C) the *k* value (x-axis) at the lowest Akaike information criterion (AIC, y-axis) represents the most parsimonious determination of the “true” *k*. B) and D) display the assignment of the *S*. *latissima* sporophytes to the optimal B) six genetic clusters (*k* = 6), and D) seven genetic clusters (*k* = 7). For B) and D) the sampling locality codes are shown on the y-axis.

**A)** **B)**


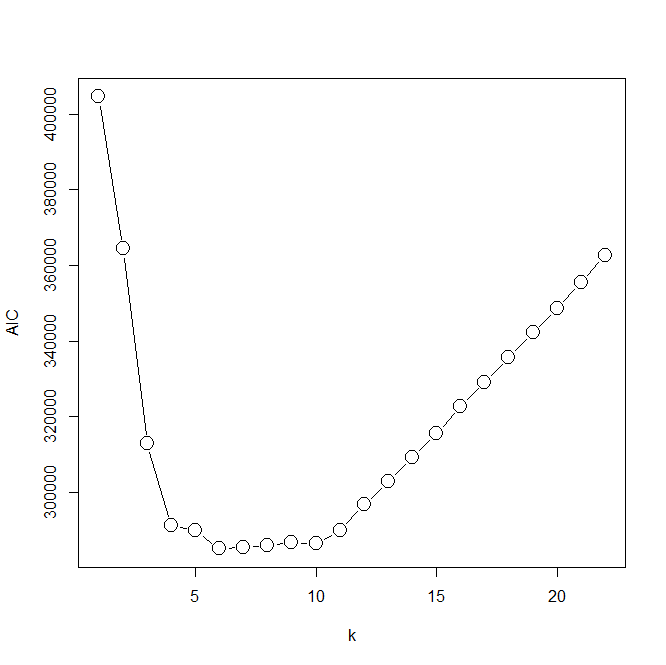

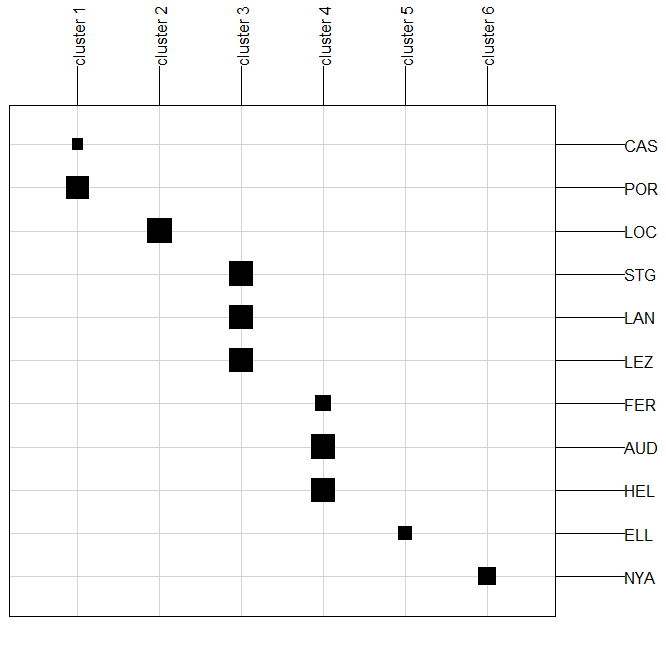


**C)** **D)**


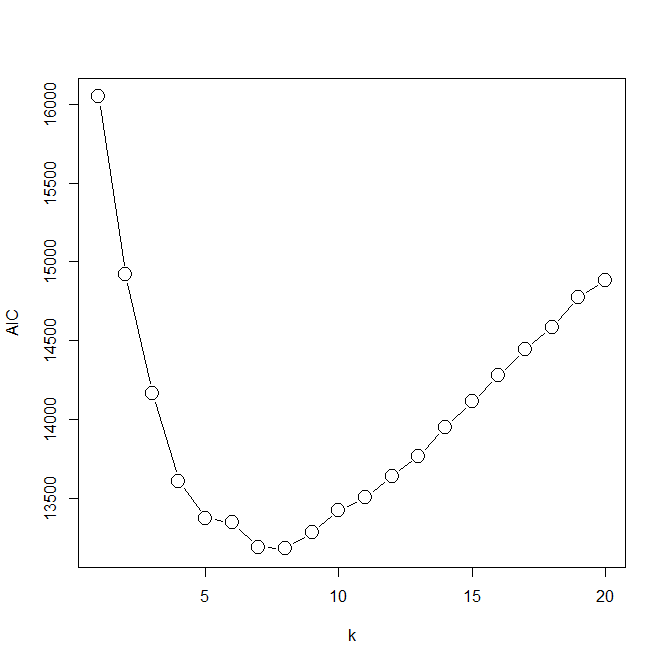

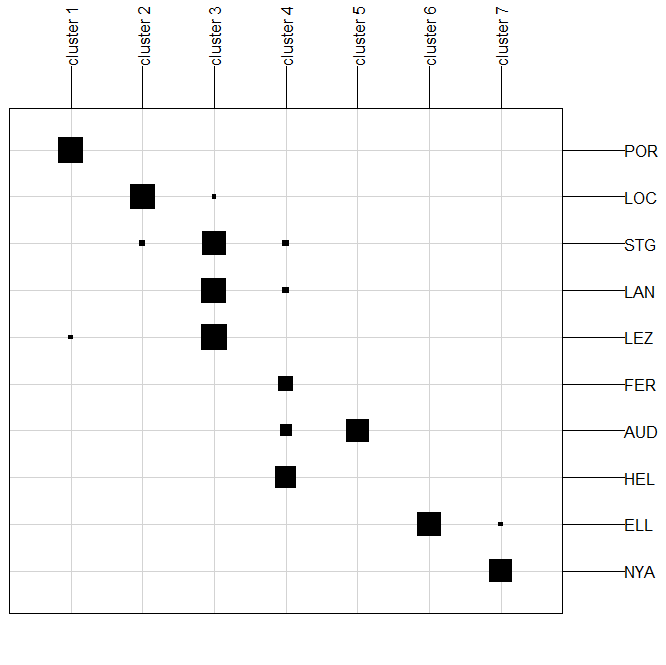


**Figure S4** DAPC scatterplot and compoplot for 199 *S*. *latissima* sporophytes sampled from 11 localities (see key) that were genotyped at 3,730 putatively neutral SNP markers. A) first two components (axes 1 and 2) of the discriminant analysis of principal components (DAPC), and B) the second and third components (axes 2 and 3) of the DAPC. Sampling localities, which were specified *a priori* for the DAPC, are differentiated by colour and inertia ellipses. Each point corresponds to a single individual. C) DAPC posterior probability of membership of each of the analysed *S*. *latissima* sporophytes (each sample is represented by a vertical bar) to each of the sampling sites (each represented by a unique colour).

**A)** **B)**


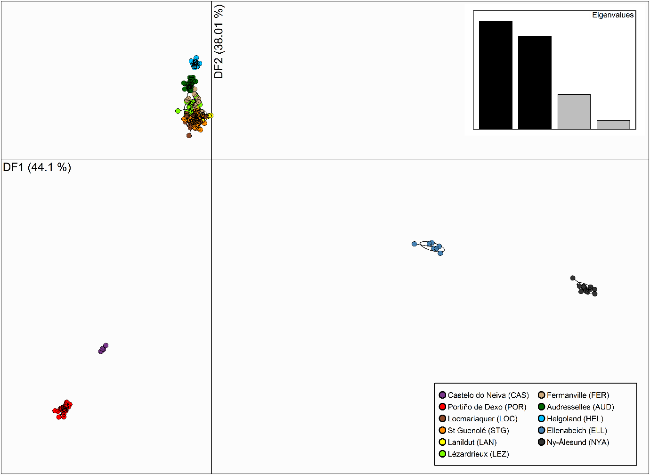

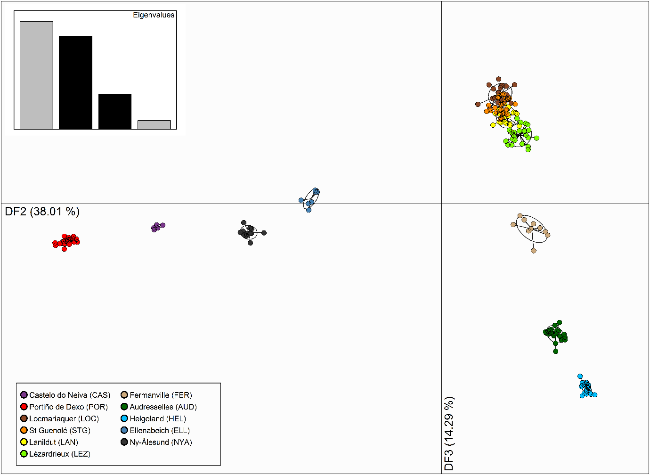


**C)**


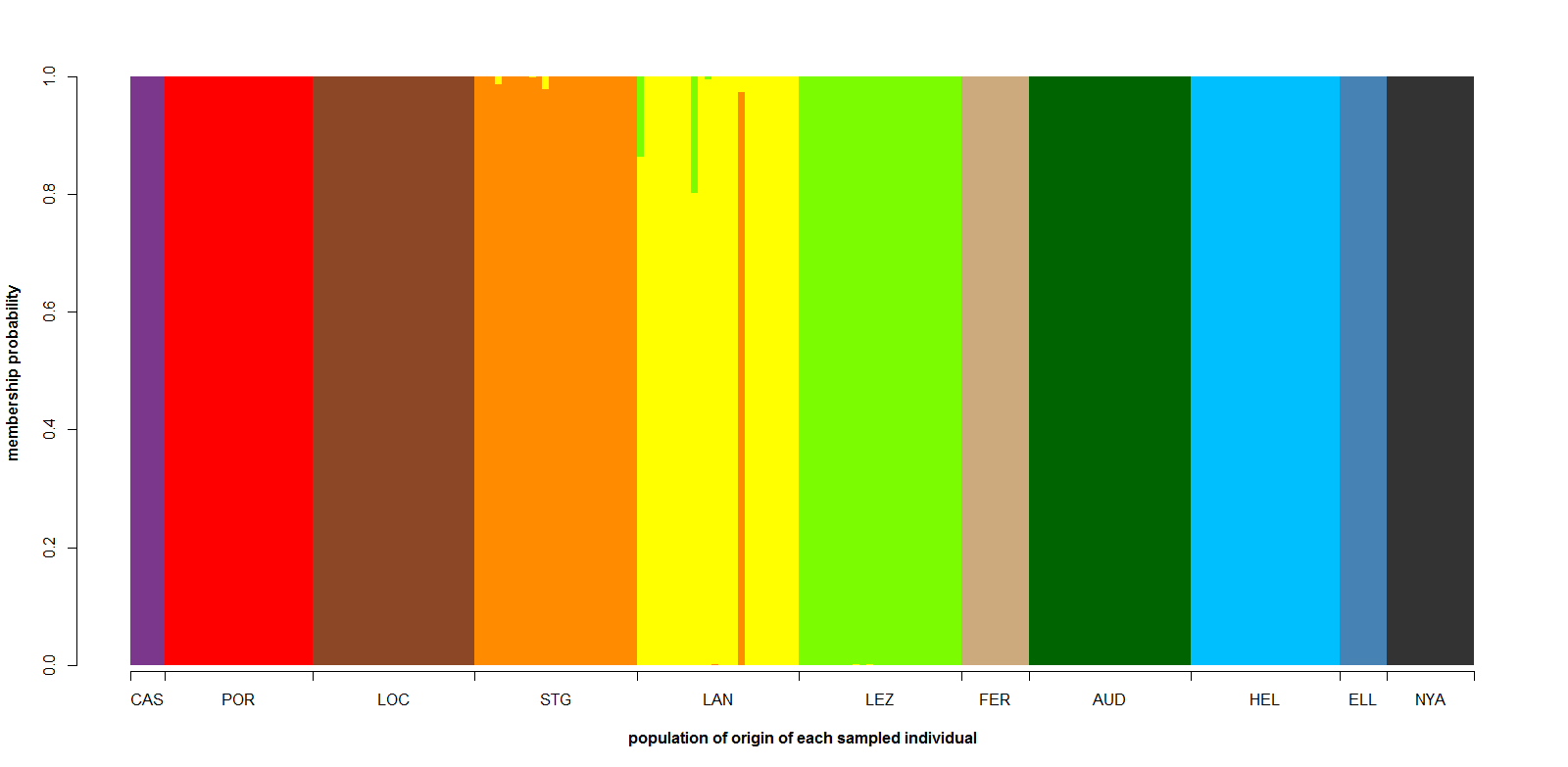


**Figure S5** DAPC scatterplot and compoplot for 280 *S*. *latissima* sporophytes sampled from 10 localities (see key) that were genotyped at 13 putatively neutral SSR markers. A) first two components (axes 1 and 2) of the discriminant analysis of principal components (DAPC), and B) the second and third components (axes 2 and 3) of the DAPC. Sampling localities, which were specified *apriori* for the DAPC, are differentiated by colour and inertia ellipses. Each point corresponds to a single individual. C) DAPC posterior probability of membership of each of the analysed *S*. *latissima* sporophytes (each sample is represented by a vertical bar) to each of the sampling sites (each represented by a unique colour).

**A)** **B)**


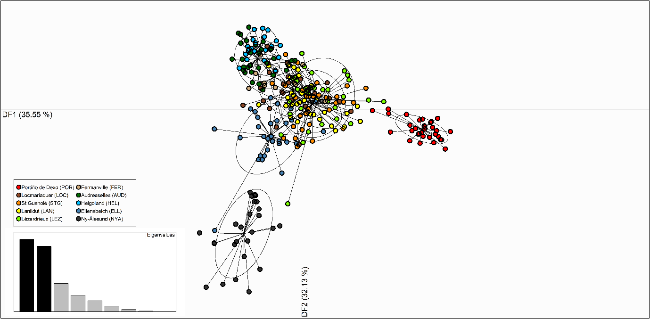

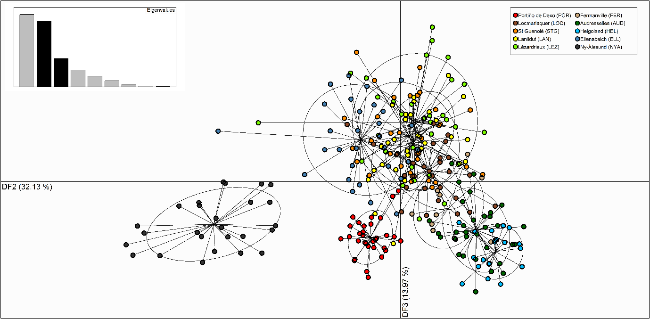


**C)**


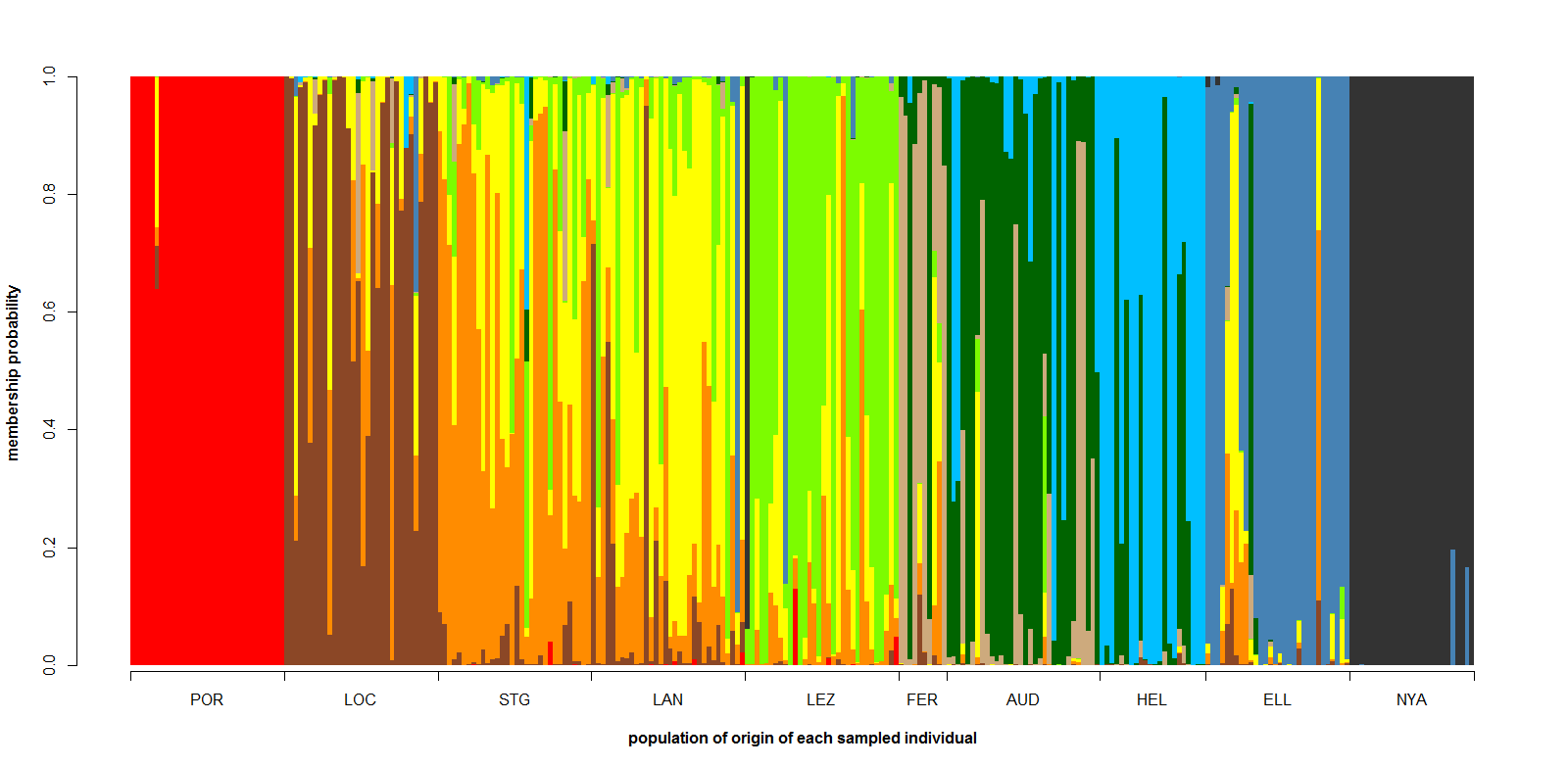


**Figure S6** Output of the *snapclust.choose.k* function of *adegenet* for 3,730 putatively neutral SNP markers that was run to determine the optimal number of genetic clusters (*k*) that best describe the genetic structure of 199 *S*. *latissima* sporophytes from 11 localities. A) the *k* value (x-axis) at the lowest Akaike information criterion (AIC, y-axis) represents the most parsimonious determination of the “true” *k*. B) the assignment of the *S*. *latissima* sporophytes to the optimal six genetic clusters (*k* = 6). Sampling locality codes are shown on the y-axis. C) Membership probability of the 199 *S*. *latissima* sporophytes (each sample is represented by a vertical bar) to each of six genetic clusters (each represented by a unique colour) that were determined as optimal by the *snapclust* algorithm run on this set of samples.

**A)** **B)**


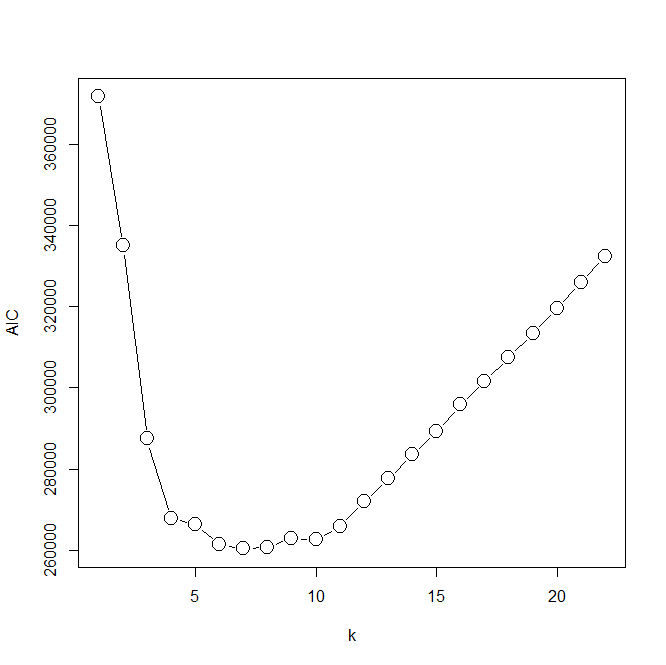

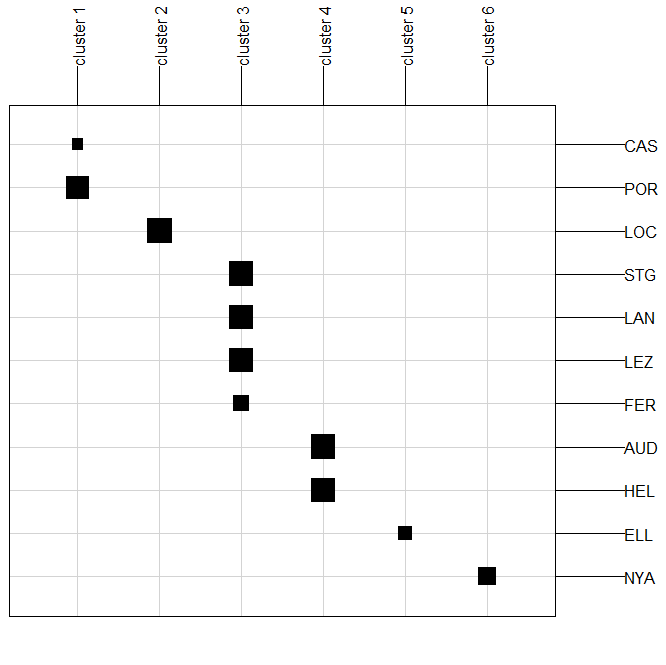


**C)**


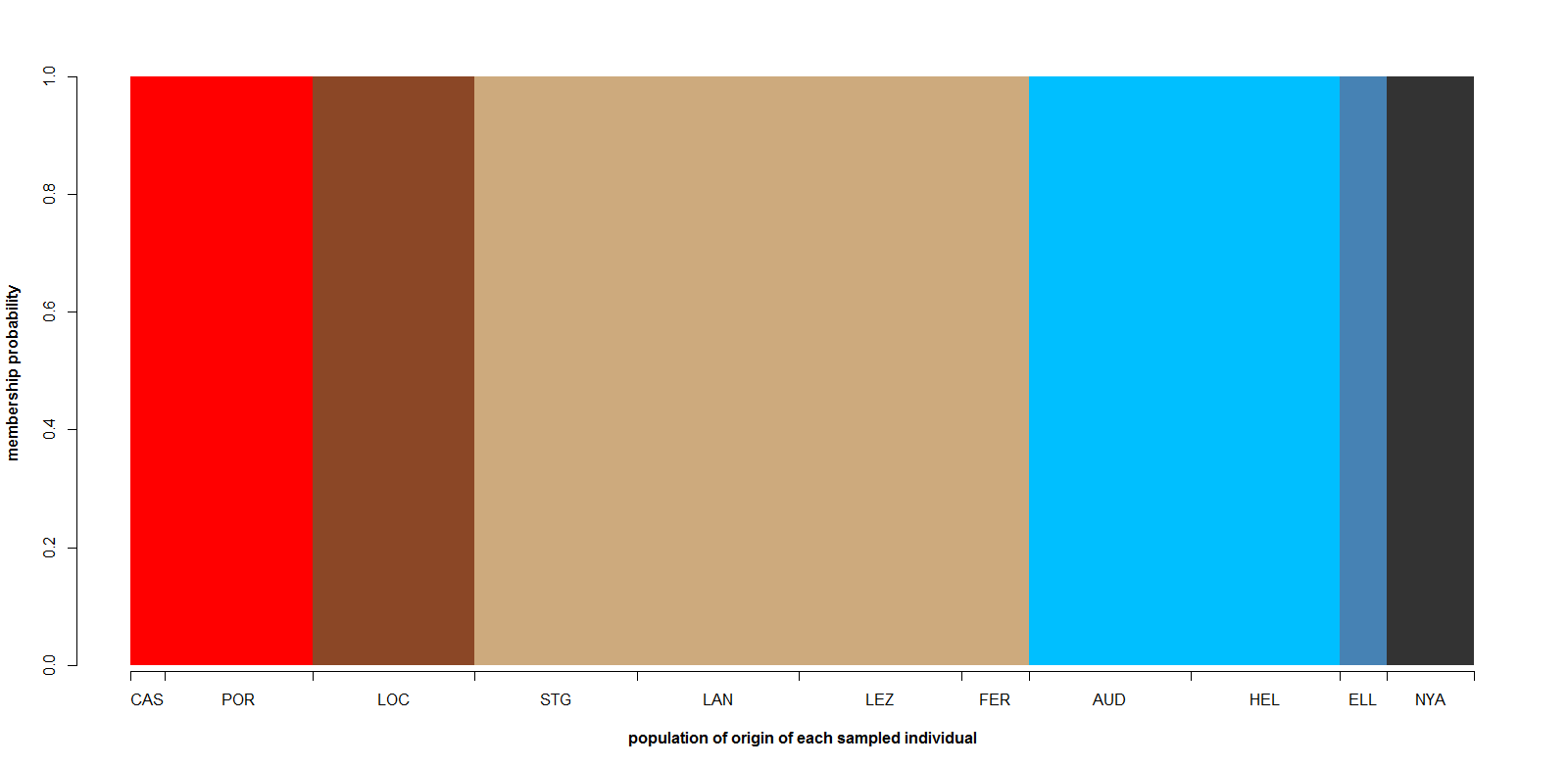


**Figure S7** Output of the *snapclust.choose.k* function of *adegenet* for 13 putatively neutral SSR markers that was run to determine the optimal number of genetic clusters (*k*) that best describe the genetic structure of 280 *S*. *latissima* sporophytes from 10 localities. A) the *k* value (x-axis) at the lowest Akaike information criterion (AIC, y-axis) represents the most parsimonious determination of the “true” *k*. B) the assignment of the *S*. *latissima* sporophytes to the optimal seven genetic clusters (*k* = 7). Sampling locality codes are shown on the y-axis. C) Membership probability of the 280 *S*. *latissima* sporophytes (each sample is represented by a vertical bar) to each of seven genetic clusters (each represented by a unique colour) that were determined by the *snapclust* algorithm run on this set of samples.

**A)** **B)**


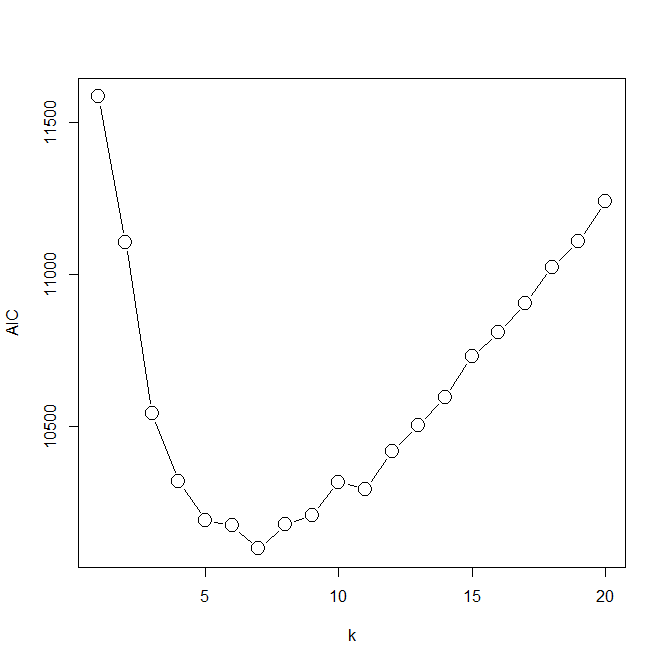

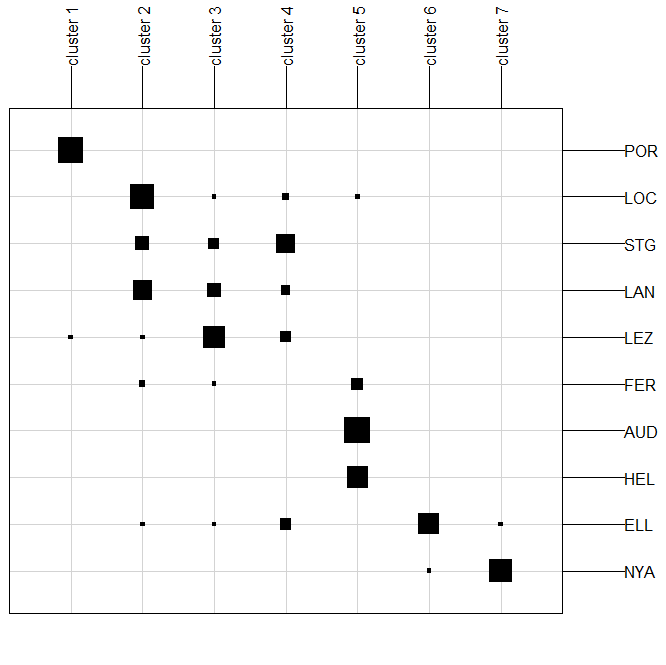


**C)**


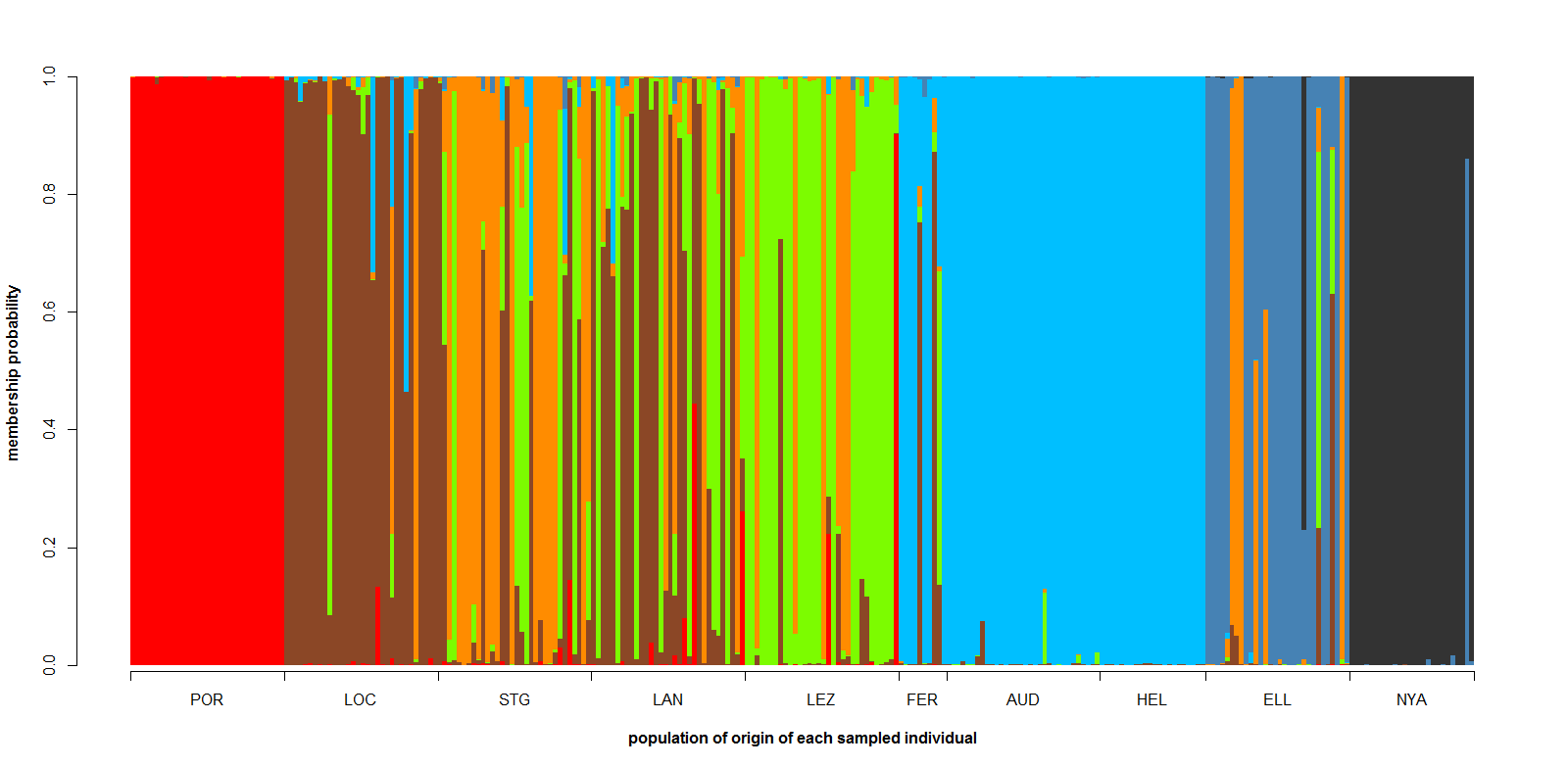


**Figure S8** Pie charts displaying the proportion of the significant nucleotide-BLAST hits of the 40 RADSeq consensus sequence tags, each harbouring a candidate outlier SNP, to A) 23 species, B) seven phyla, and C) five kingdoms.


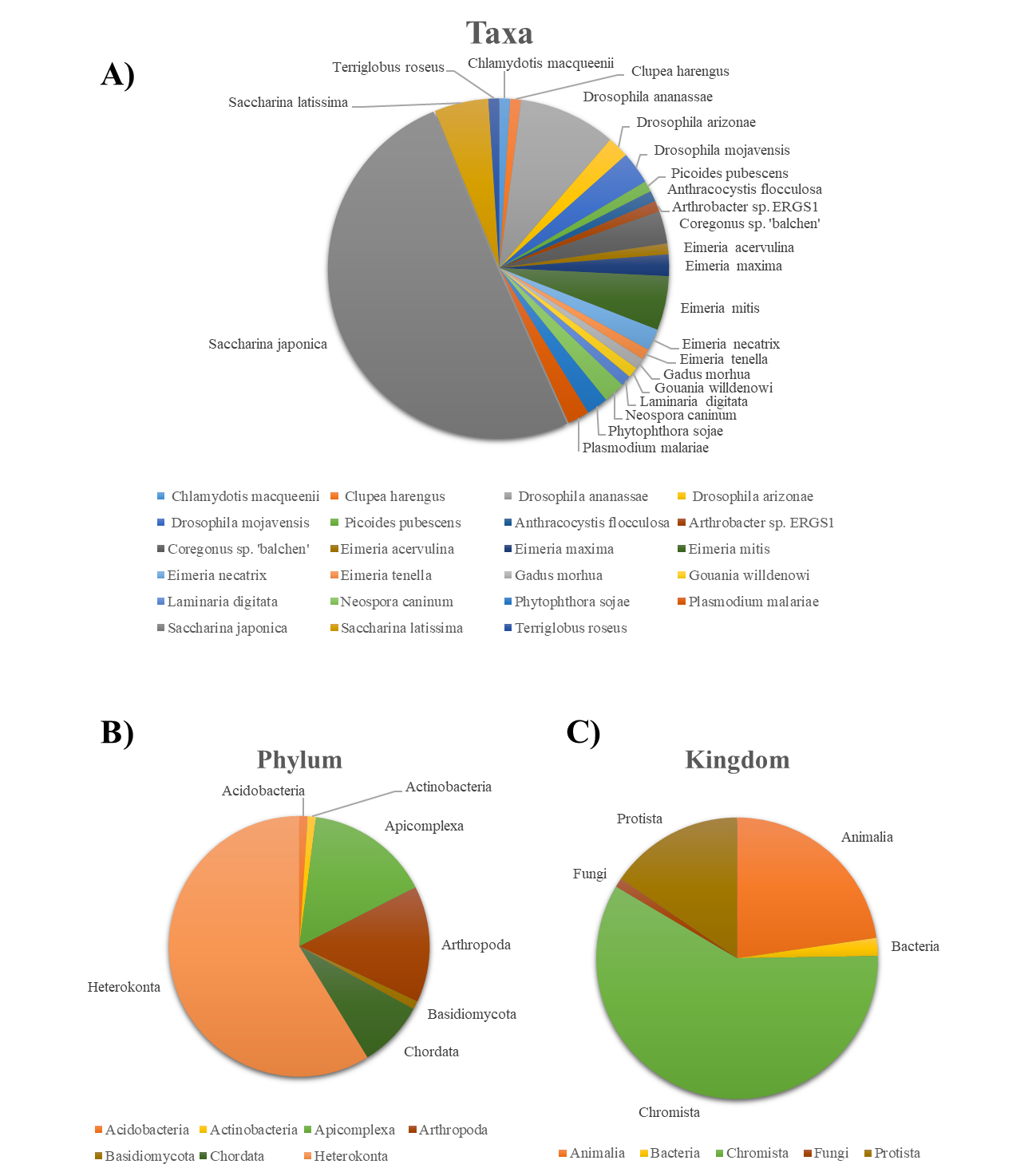

Supplement: Supplementary file 1 [file genes-11-01503-s001.zip › Supplementary Figures.docx]
