# Supplementary material for: Seascape Genomics of the Sugar Kelp Saccharina latissima along the North Eastern Atlantic Latitudinal Gradient
Source: Genes (Basel). 2020 Dec 13;11(12):1503. doi: 10.3390/genes11121503 (PMC7763533; doi:10.3390/genes11121503)
Supplement: Supplementary file 1 [file genes-11-01503-s001.zip › Supplementary Tables.docx]

**Table S1A** Description of the 11 sites where the *S*. *latissima* sporophytes were sampled for this study, including the country and geographic coordinates of each site, the names of the sample collectors, and intra-site values for 12 different environmental parameters obtained from the SeaDataNet and ERDDAP databases.

| **Site code** | **Country** | **Location name** | **Geo coord** | **Names of collectors** | **avg SST** | **min SST** | **max SST** | **c-var SST** | **avg Sal** | **min Sal** | **max Sal** | **c-var Sal** | **avg CDOM** | **min CDOM** | **max CDOM** | **c-var CDOM** |
| --- | --- | --- | --- | --- | --- | --- | --- | --- | --- | --- | --- | --- | --- | --- | --- | --- |
| CAS | Portugal | Castelo do Neiva | -8.842757E  41.621624N | R Araujo  I Azevedo | 14.74 | 13.12 | 16.90 | 0.09 | 35.37 | 35.07 | 35.64 | 0.01 | 4.93 | 1.51 | 8.21 | 0.42 |
| POR | Spain | Portiño de Dexo | -8.33264E  43.403193N | J Cremades | 15.35 | 12.78 | 18.42 | 0.15 | 35.57 | 35.48 | 35.78 | 0.00 | 3.26 | 0.36 | 6.75 | 0.66 |
| LOC | France | Locmariaquer | -2.953724E  47.491326N | M Valero  C Destombe  C Jollivet | 13.36 | 8.44 | 18.40 | 0.28 | 32.69 | 29.10 | 34.40 | 0.06 | 4.73 | 1.21 | 10.26 | 0.62 |
| STG | France | Saint Guénolé | -4.401469E  47.820255N | M Oriot | 13.33 | 9.64 | 16.79 | 0.20 | 34.91 | 34.39 | 35.28 | 0.01 | 4.50 | 0.77 | 7.90 | 0.75 |
| LAN | France | Lanildut | -4.792607E  48.479023N | J Guzinski  W Thomas | 12.50 | 9.65 | 14.74 | 0.14 | 35.08 | 34.73 | 35.40 | 0.01 | 5.10 | 1.82 | 8.40 | 0.46 |
| LEZ | France | Lézardrieux | -3.016897E  48.889614N | L Leveque | 12.66 | 9.51 | 15.40 | 0.15 | 35.12 | 34.73 | 35.60 | 0.01 | 5.40 | 1.62 | 10.59 | 0.52 |
| FER | France | Fermanville | -1.486632E  49.726228N | S Mauger  J Guzinski  W Thomas | 12.44 | 9.90 | 16.90 | 0.21 | 34.46 | 33.50 | 35.17 | 0.02 | 5.14 | 1.03 | 8.56 | 0.48 |
| AUD | France | Audresselles | 1.499115E  50.851796N | C Jollivet  J Coudret  B Jacquemin | 11.88 | 7.34 | 17.76 | 0.33 | 34.17 | 33.55 | 34.82 | 0.01 | 6.38 | 3.20 | 8.53 | 0.31 |
| HEL | Germany | Helgoland | 7.869016E  54.19193N | I Bartsch | 10.19 | 4.65 | 17.27 | 0.46 | 31.28 | 30.28 | 32.61 | 0.02 | 5.15 | 1.84 | 8.33 | 0.50 |
| ELL | Scotland | Ellenabeich | -5.734236E  56.307873N | A Peters | 10.06 | 6.24 | 13.56 | 0.23 | 32.39 | 31.66 | 32.94 | 0.01 | 3.88 | 0.84 | 7.73 | 0.53 |
| NYA | Norway | Ny-Ålesund | 9.007976E  79.333862N | I Bartsch | 2.72 | 0.23 | 6.37 | 0.75 | 33.75 | 32.72 | 34.32 | 0.02 | 1.28 | 0.23 | 2.94 | 0.57 |

avg SST = average sea surface temperature (in degrees Celsius); min SST = minimum sea surface temperature detected in a single year (in degrees Celsius); max SST = maximum sea surface temperature detected in a single year (in degrees Celsius); c-var SST = coefficient of variation of sea surface temperature detected in a single year; avg Sal = average salinity (in psu); min Sal = minimum salinity detected in a single year (in psu); max Sal = maximum salinity detected in a single year (in psu); c-var SST = coefficient of variation of salinity; avg CDOM = average chromophoric dissolved organic material (absorbance at 412 nm); min CDOM = minimum chromophoric dissolved organic material (absorbance at 412 nm); max CDOM = maximum chromophoric dissolved organic material (absorbance at 412 nm); c-var CDOM = coefficient of variation of chromophoric dissolved organic material in a single year.

**Table S1B** Pairwise correlation among the 12 environmental parameters obtained from the SeaDataNet and ERDDAP databases. P-values after Bonferroni correction shown above the diagonal. P-values in bold indicate significant correlation among the environmental parameters. Pearson`s correlation coefficient (r) shown below the diagonal.

|  | **avg SST** | **min SST** | **max SST** | **c-var SST** | **avg**  **Sal** | **min**  **Sal** | **max**  **Sal** | **c-var**  **Sal** | **avg CDOM** | **min CDOM** | **max CDOM** | **c-var CDOM** |
| --- | --- | --- | --- | --- | --- | --- | --- | --- | --- | --- | --- | --- |
| **avg SST** |  | **0.0007** | **0.0115** | **0.0063** | 1.000 | 1.000 | 1.000 | 1.000 | 1.000 | 1.000 | 0.9345 | 1.000 |
| **min SST** | 0.9458 |  | 0.7069 | **0.0022** | 1.000 | 1.000 | 1.000 | 1.000 | 1.000 | 1.000 | 1.000 | 1.000 |
| **max SST** | 0.8980 | 0.7303 |  | 1.000 | 1.000 | 1.000 | 1.000 | 1.000 | 0.6558 | 1.000 | 0.4905 | 1.000 |
| **c-var SST** | -0.9113 | -0.9298 | -0.6880 |  | 1.000 | 1.000 | 1.000 | 1.000 | 1.000 | 1.000 | 1.000 | 1.000 |
| **avg**  **Sal** | 0.4304 | 0.6495 | 0.0920 | -0.5206 |  | **0.0036** | **0.0001** | 1.000 | 1.000 | 1.000 | 1.000 | 1.000 |
| **min**  **Sal** | 0.3314 | 0.5527 | 0.0143 | -0.4684 | 0.9216 |  | 0.2253 | 0.2170 | 1.000 | 1.000 | 1.000 | 1.000 |
| **max**  **Sal** | 0.5042 | 0.6852 | 0.2104 | -0.5254 | 0.9623 | 0.7955 |  | 1.000 | 1.000 | 1.000 | 1.000 | 1.000 |
| **c-var**  **Sal** | -0.1020 | -0.2441 | 0.0806 | 0.2587 | -0.5083 | -0.7973 | -0.2811 |  | 1.000 | 1.000 | 1.000 | 1.000 |
| **avg CDOM** | 0.6182 | 0.4575 | 0.7352 | -0.5643 | 0.0334 | 0.0281 | 0.1176 | 0.0143 |  | 0.0955 | 0.0660 | 1.000 |
| **min CDOM** | 0.2333 | 0.0826 | 0.4088 | -0.1635 | -0.0777 | -0.0289 | -0.0291 | -0.0301 | 0.8332 |  | 1.000 | 0.3374 |
| **max CDOM** | 0.7111 | 0.5432 | 0.7533 | -0.6813 | -0.0092 | -0.1215 | 0.1278 | 0.2635 | 0.8470 | 0.5217 |  | 1.000 |
| **c-var CDOM** | 0.0472 | 0.0389 | -0.0386 | 0.0146 | 0.03627 | -0.0654 | 0.0599 | 0.1524 | -0.5164 | -0.7747 | -0.1730 |  |

avg SST = average sea surface temperature (in degrees Celsius); min SST = minimum sea surface temperature detected in a single year (in degrees Celsius); max SST = maximum sea surface temperature detected in a single year (in degrees Celsius); c-var SST = coefficient of variation of sea surface temperature detected in a single year; avg Sal = average salinity (in psu); min Sal = minimum salinity detected in a single year (in psu); max Sal = maximum salinity detected in a single year (in psu); c-var SST = coefficient of variation of salinity; avg CDOM = average chromophoric dissolved organic material (absorbance at 412 nm); min CDOM = minimum chromophoric dissolved organic material (absorbance at 412 nm); max CDOM = maximum chromophoric dissolved organic material (absorbance at 412 nm); c-var CDOM = coefficient of variation of chromophoric dissolved organic material in a single year.

**Table S2A** Pairwise comparison of the within-locality *H_E_* (in brackets) amongst 11 *S*. *latissima* localities genotyped with SNPs. Shown are the FDR corrected Welch Two Sample t-test p-values. Values in red indicate significance after FDR correction (at a false discovery rate of 0.05).

|  | **CAS (0.034)** | **POR (0.036)** | **LOC (0.051)** | **STG (0.054)** | **LAN (0.052)** | **LEZ (0.051)** | **FER (0.045)** | **AUD (0.027)** | **HEL (0.014)** | **ELL (0.064)** | **NYA (0.060)** |
| --- | --- | --- | --- | --- | --- | --- | --- | --- | --- | --- | --- |
| **CAS (0.034)** | NA |  |  |  |  |  |  |  |  |  |  |
| **POR (0.036)** | 0.455 | NA |  |  |  |  |  |  |  |  |  |
| **LOC (0.051)** | 0.000 | 0.000 | NA |  |  |  |  |  |  |  |  |
| **STG (0.054)** | 0.000 | 0.000 | 0.162 | NA |  |  |  |  |  |  |  |
| **LAN (0.052)** | 0.000 | 0.000 | 0.480 | 0.492 | NA |  |  |  |  |  |  |
| **LEZ (0.051)** | 0.000 | 0.000 | 0.960 | 0.159 | 0.468 | NA |  |  |  |  |  |
| **FER (0.045)** | 0.000 | 0.000 | 0.030 | 0.000 | 0.004 | 0.037 | NA |  |  |  |  |
| **AUD (0.027)** | 0.005 | 0.000 | 0.000 | 0.000 | 0.000 | 0.000 | 0.000 | NA |  |  |  |
| **HEL (0.014)** | 0.000 | 0.000 | 0.000 | 0.000 | 0.000 | 0.000 | 0.000 | 0.000 | NA |  |  |
| **ELL (0.064)** | 0.000 | 0.000 | 0.000 | 0.001 | 0.000 | 0.000 | 0.000 | 0.000 | 0.000 | NA |  |
| **NYA (0.060)** | 0.000 | 0.000 | 0.002 | 0.067 | 0.015 | 0.002 | 0.000 | 0.000 | 0.000 | 0.159 | NA |

**Table S2B** Pairwise comparison of the within-locality *H_E_* (in brackets) amongst 10 *S*. *latissima* localities genotyped with SSRs. Shown are the FDR corrected Welch Two Sample t-test p-values. Values in red indicate comparisons that were significant before FDR correction (at a value of alpha of 0.05).

|  | **POR (0.228)** | **LOC (0.231)** | **STG (0.316)** | **LAN (0.289)** | **LEZ (0.330)** | **FER (0.216)** | **AUD (0.249)** | **HEL (0.181)** | **ELL (0.382)** | **NYA (0.367)** |
| --- | --- | --- | --- | --- | --- | --- | --- | --- | --- | --- |
| **POR (0.228)** | NA |  |  |  |  |  |  |  |  |  |
| **LOC (0.231)** | 0.977 | NA |  |  |  |  |  |  |  |  |
| **STG (0.316)** | 0.888 | 0.888 | NA |  |  |  |  |  |  |  |
| **LAN (0.289)** | 0.888 | 0.888 | 0.923 | NA |  |  |  |  |  |  |
| **LEZ (0.330)** | 0.888 | 0.888 | 0.923 | 0.888 | NA |  |  |  |  |  |
| **FER (0.216)** | 0.923 | 0.923 | 0.888 | 0.888 | 0.888 | NA |  |  |  |  |
| **AUD (0.249)** | 0.923 | 0.923 | 0.888 | 0.888 | 0.888 | 0.888 | NA |  |  |  |
| **HEL (0.181)** | 0.888 | 0.888 | 0.809 | 0.888 | 0.809 | 0.888 | 0.888 | NA |  |  |
| **ELL (0.382)** | 0.809 | 0.809 | 0.888 | 0.888 | 0.888 | 0.809 | 0.809 | 0.809 | NA |  |
| **NYA (0.367)** | 0.809 | 0.809 | 0.888 | 0.888 | 0.888 | 0.809 | 0.809 | 0.809 | 0.923 | NA |

**Table S3** Pearson's product-moment correlation of *H_E_*, allelic richness, and percentage of private alleles with latitude for 11 (marker type – SNP) or 10 sampling sites (marker type –SSR).

| **Genetic diversity index (marker type)** | **Correlation coefficient** | **P-value** |
| --- | --- | --- |
| ***H_E_* (SNP)** | 0.339 | 0.308 |
| **Allelic richness (SNP)** | 0.244 | 0.429 |
| **Private allele % (SNP)** | 0.531 | 0.093 |
| ***H_E_* (SSR)** | 0.507 | 0.135 |
| **Allelic richness (SSR)** | 0.063 | 0.864 |
| **Private allele % (SSR)** | 0.256 | 0.475 |

**Table S4** Pairwise *F*_ST_ for the 11 *S*. *latissima* localities that were compared at the European geographic scale. Data are presented for the RAD dataset (4,069 SNPs) – below the diagonal, and for the SSR dataset (18 SSR loci) – above the diagonal. All values were significant at alpha of 0.001. FDR correction was applied.

|  | **CAS** | **POR** | **LOC** | **STG** | **LAN** | **LEZ** | **FER** | **AUD** | **HEL** | **ELL** | **NYA** |
| --- | --- | --- | --- | --- | --- | --- | --- | --- | --- | --- | --- |
| **CAS** | 0 | NA | NA | NA | NA | NA | NA | NA | NA | NA | NA |
| **POR** | 0.463 | 0 | 0.243 | 0.206 | 0.232 | 0.223 | 0.375 | 0.339 | 0.401 | 0.287 | 0.363 |
| **LOC** | 0.509 | 0.508 | 0 | 0.099 | 0.119 | 0.150 | 0.281 | 0.193 | 0.314 | 0.207 | 0.300 |
| **STG** | 0.488 | 0.491 | 0.127 | 0 | 0.036 | 0.059 | 0.186 | 0.167 | 0.281 | 0.179 | 0.270 |
| **LAN** | 0.502 | 0.506 | 0.169 | 0.060 | 0 | 0.041 | 0.155 | 0.156 | 0.256 | 0.208 | 0.311 |
| **LEZ** | 0.523 | 0.526 | 0.196 | 0.121 | 0.108 | 0 | 0.180 | 0.164 | 0.263 | 0.203 | 0.297 |
| **FER** | 0.574 | 0.573 | 0.263 | 0.201 | 0.200 | 0.185 | 0 | 0.200 | 0.213 | 0.266 | 0.328 |
| **AUD** | 0.684 | 0.642 | 0.369 | 0.320 | 0.321 | 0.315 | 0.297 | 0 | 0.250 | 0.269 | 0.342 |
| **HEL** | 0.796 | 0.718 | 0.468 | 0.426 | 0.422 | 0.429 | 0.449 | 0.397 | 0 | 0.340 | 0.401 |
| **ELL** | 0.559 | 0.597 | 0.434 | 0.407 | 0.416 | 0.446 | 0.469 | 0.601 | 0.697 | 0 | 0.171 |
| **NYA** | 0.611 | 0.636 | 0.515 | 0.496 | 0.502 | 0.526 | 0.543 | 0.642 | 0.708 | 0.355 | 0 |

**Table S5** Candidate outlier SSR loci alleles that correlated significantly with 12 environmental parameters in a pairwise comparison test.

| **Locus/allele** | **Species associated with Genbank hits** | **Genome location** | **Environmental correlations** |
| --- | --- | --- | --- |
| Sacl33-182 (microsatellite) | *Saccharina latissima* | EST-derived microsatellite sequence | Min CMOD (+) [R^2^ = 0.60] |
| Sacl33-186 (microsatellite) | *Saccharina latissima* | EST-derived microsatellite sequence | Min CMOD (-) [R^2^ = 0.58] |
| Sacl56-111 (microsatellite) | *Saccharina latissima* | EST-derived microsatellite sequence | Average SST (-) [R^2^ = 0.79]  MIn SST (-) [R^2^ = 0.56]  Max SST (-) [R^2^ = 0.78]  cvar SST (+) [R^2^ = 0.71]  Average CDOM (-) [R^2^ = 0.59]  Max CDOM (-) [R^2^ = 0.70] |
| Sacl56-115 (microsatellite) | *Saccharina latissima* | EST-derived microsatellite sequence | Average SST (+) [R^2^ = 0.64]  cvar SST (-) [R^2^ = 0.71]  Max CDOM (+) [R^2^ = 0.67] |
| Sacl60-182 (microsatellite) | *Saccharina latissima* | EST-derived microsatellite sequence | Min CDOM (+) [R^2^ = 0.59]  cvar CDOM (-) [R^2^ = 0.45] |
| Sacl88-147 (microsatellite) | *Saccharina latissima* | EST-derived microsatellite sequence | Max SST (-) [R^2^ = 0.63] |
| SLN32-227 (microsatellite) | *Saccharina latissima* | Genomic microsatellite sequence | Average SST (-) [R^2^ = 0.64]  Max SST (-) [R^2^ = 0.75] |
| SLN32-245 (microsatellite) | *Saccharina latissima* | Genomic microsatellite sequence | Average SST (-) [R^2^ = 0.76]  Min SST (-) [R^2^ = 0.59]  Max SST (-) [R^2^ = 0.74]  cvar SST (+) [R^2^ = 0.73]  Average CDOM (-) [R^2^ = 0.59]  Max CDOM (-) [R^2^ = 0.61] |
| SLN32-254 (microsatellite) | *Saccharina latissima* | Genomic microsatellite sequence | Average SST (-) [R^2^ = 0.64]  Max SST (-) R^2^ = 0.72]  Average CDOM (-) R^2^ = 0.63]  Max CDOM (-) R^2^ = 0.66] |
| SLN32-266 (microsatellite) | *Saccharina latissima* | Genomic microsatellite sequence | cvar Salinity (+) [R^2^ = 0.63] |
| SLN54-349 (microsatellite) | *Saccharina latissima* | Genomic microsatellite sequence | Average SST (-) [R^2^ = 0.82]  Min SST (-) [R^2^ = 0.61]  Max SST (-) [R^2^ = 0.83]  cvar SST (+) [R^2^ = 0.73]  Average CDOM (-) [R^2^ = 0.65]  Max CDOM (-) [R^2^ = 0.72] |
| SLN320-219 (microsatellite) | *Saccharina latissima* | Genomic microsatellite sequence | Average Salinity (-) [R^2^ = 0.45]  Max Salinity (-) [R^2^ = 0.42] |

Average SST = average sea surface temperature (in degrees Celsius); Min SST = minimum sea surface temperature detected in a single year (in degrees Celsius); Max SST = maximum sea surface temperature detected in a single year (in degrees Celsius); cvar SST = coefficient of variation of sea surface temperature detected in a single year; Average Salinity = average salinity (in psu); Min Salinity = minimum salinity detected in a single year (in psu); Max Salinity = maximum salinity detected in a single year (in psu); cvar Salinity = coefficient of variation of salinity; Average CDOM = average chromophoric dissolved organic material (absorbance at 412 nm); Min CDOM = minimum chromophoric dissolved organic material (absorbance at 412 nm); Max CDOM = maximum chromophoric dissolved organic material (absorbance at 412 nm); cvar CDOM = coefficient of variation of chromophoric dissolved organic material in a single year. (+) = positive correlation; (-) = negative correlation. R^2^ = coefficient of determination.
