# Supplementary material for: Seascape Genomics of the Sugar Kelp Saccharina latissima along the North Eastern Atlantic Latitudinal Gradient
Source: Genes (Basel). 2020 Dec 13;11(12):1503. doi: 10.3390/genes11121503 (PMC7763533; doi:10.3390/genes11121503)
Supplement: Supplementary file 1 [file genes-11-01503-s001.zip › Supplementary Text.docx]

**Supplementary Text**

**Materials & Methods**

*Laboratory preparation and sequencing of ddRAD-seq libraries*

Laboratory preparation of *S*. *latissima* sporophyte samples for double-digest RAD sequencing (ddRAD-seq) was performed as detailed in Guzinski et al. (2018). The initial aim was to sequence eight ddRAD-seq libraries, one library per lane of a HiSeq flow-cell, with each library comprising 84 samples. The eight libraries were all constructed in the same manner, sequentially (i.e. library 1 to library 8), and independently (there was no overlap of samples between the libraries except for three samples sequenced as controls in each of the libraries). Briefly, DNA extracted from dry sporophyte tissue was digested with a pair of restriction enzymes, *Pst*I and *Mse*I. For each of the samples, the completeness of the double digest reaction was assessed by separating the digested DNA by agarose gel electrophoresis. Next, the P1 and P2 adapters were ligated to the digested DNA fragments. The P1 adapters carried 21 unique barcodes, and as each sample had its digested DNA annealed to adapters with a single barcode only, four samples per library were marked with the same barcode. After purification with the magnetic beads, the DNA of each of the samples was PCR amplified with primers complementary to the adapters flanking each DNA fragment. The PCR primer sequences were indexed with one of four unique indexes. Amplifying each of the four samples that were tagged with the same adapter barcode with a uniquely indexed PCR primer pair allowed to multiplex all 84 samples comprising a library. PCR amplification strength was assessed visually for each of the samples via agarose gel electrophoresis. Samples that amplified better were added in a lesser volume to the library pool compared to the weaker amplified samples, with the aim of attaining equimolar concentrations for all of the pooled samples. Next, the pooled libraries were size selected for fragments between 300 and 650 bp using a Pippin Prep 1.5% cassette. Prior to sending the size-selected libraries to the sequencing facility, the libraries were analyzed using a High Sensitivity DNA chip on a Bioanalyser Agilent2100 instrument, and quantified with qPCR (Kapa Biosystems qPCR kit) and a Qubit assay (high sensitivity kit).

However many of the samples that were supposed to be included in the initial eight libraries failed to amplify at the PCR stage of the library construction. These amplification failures most likely were due to the polysaccharide inhibition of the PCR. Despite several retrials, many of the samples had to be abandoned or replaced with samples belonging to other populations. This, coupled with issues related to obtaining a sufficiently high number of reads for many of the samples that were included in two of the libraries sequenced at Fasteris, meant that in total we sequenced 10 libraries comprising a total of 544 samples. Some of the samples, i.e. those that sequenced poorly in the initial sequencing effort, were sequenced twice. Therefore, the adapter barcode and primer index labelling of the samples could not always be carried out as described above, with several additional barcodes and indices necessary to multiplex the samples making up the ten libraries.

*Processing of the ddRAD-seq genotyped samples*

Of these 544 samples, all of which were part of a substantial sampling and sequencing effort, only 199 sporophytes collected from 11 localities were relevant to the current study. Post-sequencing all of the 544 samples were processed together, and below we describe the most relevant steps for the filtering of the reads against several quality control criteria for this whole larger dataset.

After sequencing the reads were first demultiplexed by the index, which was performed at Fasteris, and subsequently by the barcode, utilising the *process_radtags* function of Stacks 1.40 (Catchen et al. 2013). For those individuals that were sequenced twice because of poor initial sequencing effort, reads were combined from both sequencing efforts prior to further processing of reads from those samples. In total, there were 1,294,743,101 reads - on average 2,380,042 reads for each of the 544 individuals. Before processing the reads further, cutadapt 1.8.3 (Martin 2011) was used to remove reads contaminated with the adapter sequence (14.6% reads removed) and to trim the 5′ *Pst*I overhang nucleotides from the beginning of each of the remaining reads. Locus assembly and single nucleotide polymorphism (SNP) calling was carried out with the *denovo_map.pl* wrapper in Stacks. The *denovo_map.pl* parameters were selected such that the risk of assembling non-homologous loci was minimized. The –m parameter (minimal stack depth) was set to five, the –M parameter (maximum number of allowed mismatches within each locus at the individual sample level) was set to four, the –n parameter (maximum number of allowed mismatches when merging loci across the 544 sample dataset) was set to six in order to account for the wide, pan-European sampling range, and the –N parameter (maximum number of mismatches allowed when aligning the secondary reads to the primary stacks) was set to two. Employing this set of parameters produced a 544-sample catalog that was populated with 1,377,662 loci. These were reduced to 13,122 polymorphic loci (featuring 103,963 SNPs) after setting the –r filter of the Stacks *populations* function to 0.9, thus retaining only those loci present in 90% of the 544 samples. Quality of SNP calling was assessed by comparing genotypes across replicates (three samples that were present in all of the sequenced libraries), with genotype congruence averaging 99.7%. All further dataset manipulation and filtering was performed in base R or in a specific R package on the variant calling format (VCF) file exported from *populations*. The dataset was subsequently reduced to 458 samples after removal of the replicates, those individuals that exhibited in excess of 10% missing data, and three samples that were misidentified in the field as *S*. *latissima* but in fact were *Laminaria digitata*. Next, in order to minimise linkage disequilibrium in the dataset, we randomly retained only a single SNP from each of the polymorphic reads. This resulted in a 13,122 SNP marker set.

*SNP filtering on the 199-sample dataset*

Further filtering of the SNP markers was performed on the 199 samples (collected from 11 localities spanning the European Atlantic coast range of *S*. *latissima*) relevant for the study described herein. We used the filter_maf function of the *radiator* R package (Gosselin et al. 2020) to perform minor allele frequency (MAF) selection on the 7,511 ddRAD-seq markers that were polymorphic after the dataset was reduced to 199 individuals. A locus was purged from the dataset if at the within-locality level its MAF was below 0.04 (thus a locus was retained if the alternative (i.e. minor) allele occurred at least approximately two times out of 48 times (as the median number of individuals in localities with ten or more individuals per locality was 24)) or if its overall or global MAF was below 0.01 (thus a locus was retained if the alternative allele occurred at least approximately five times out of 498 times (199 diploid individuals*2)). As several localities comprised less than ten individuals (Table 1), SNPs were removed from the dataset even if they passed the local MAF criterion, but only in one (or more) of these low sample number localities. The final set of SNPs comprised 4,069 markers originating from 4,069 loci. Overall the dataset exhibited 2.41% missing data. A single individual had in excess of 10% of missing data (10.35%), and 36 individuals had in excess of 5% of missing data as revealed by analysis in R package *grur* (Gosselin 2018). Additionally, 73 loci had in excess of 10% of missing data, with maximum of 16.08% missing data at two loci. These patterns of missing data prompted us to impute the missing data utilising a random forest machine learning algorithm implemented through the genomic_converter function of the *radiator* R package. Imputations were performed independently within each of the 11 localities (hierarchical.levels = "strata"). The num.tree argument was set to 100, otherwise the arguments were set to default values. Proportion of missing genotypes after imputations was 0.14% as the missing data for those loci for which there was missing data for all individuals within a locality could not be imputed. To facilitate the utilisation of this dataset for the analyses in various software, conversion of data from VCF to different input formats was performed in R or in PGDSpider (Lischer & Excoffier 2012).

*Microsatellite (SSR) genotyping*

The initial aim was to utilise 12 expressed sequence tag (EST)-derived SSR loci from Guzinski et al. (2016) and eight genomic SSR loci from Paulino et al. (2016) as the SSR genetic markers used in our study, however two EST-derived SSR loci (Sacl-08 and Sacl-27) were removed from the final set of loci due to difficulties in reliably scoring a locus and due to a locus successfully amplifying only in certain populations, respectively. Thus the final panel of 18 SSR loci used in the study comprised:

Sacl-13, Sacl-21, Sacl-32, Sacl-33, Sacl-41, Sacl-56, Sacl-60, Sacl-75, Sacl-78, Sacl-88 ten EST-derived SSR loci, and SLN32, SLN34, SLN35, SLN36, SLN54, SLN319, SLN320, SLN510 eight genomic SSR loci.

**Results**

*Population genetic structure based on neutral SNP and SSR markers*

Population structure assessed after removal of the putative outlier SNPs, such that 3,730 SNPs were remaining, was broadly similar to the results obtained with the full set of SNPs, especially for the DAPC. There was very high similarity for the full SNP set and the neutral SNP set for the DAPC outputs comprising the first two components of the DAPC (compare Figure 2A and Figure S4A), the second and the third components of the DAPC (compare Figure S2A and S4B), and the compoplot (compare Figure 2C and S4C). The optimal *k* for the snapclust algorithm ran on the neutral-only SNP set was six (Figure S6A). The composition of the six resulting genetic clusters (Figure S6B, Figure S6C) was identical to the composition of the six genetic clusters suggested by snapclust for the full locus set (Figure S3B, Figure 3A), except that the FER locality was grouped with the Brittany localities of STG, LAN, LEZ and not with the English Channel/North Sea localities of AUD and HEL. Similarly, the DAPC outputs for the 13 neutral SSR loci closely resembled those obtained for the full SSR locus set: compare the outputs for the DAPC axes 1 and 2 (Figure 2B and Figure S5A), the DAPC axes 2 and 3 (Figure S2B and Figure S5B), and the compoplot (Figure 2D and Figure S5C). Utilising the 13 neutral SSR loci to assess the population genetic structure at the ten localities with the *snapclust* algorithm revealed that the optimal *k* was seven (Figure S7A), which was also the optimal *k* selected for the full SSR locus set (Figure S3C). However the clustering patterns obtained with the neutral-only (Figure S7B and Figure S7C) and full (Figure S3D and Figure 3B) SSR locus sets were only broadly congruent. With the neutral-only locus set there was substantially greater separation amongst the Brittany localities (STG, LAN, LEZ), which formed a largely contiguous single cluster for the full locus set, and the AUD locality was clearly integrated with the FER and HEL localities as part of a single cluster.

**References**

[Catchen](https://onlinelibrary.wiley.com/action/doSearch?ContribAuthorStored=Catchen%2C+Julian), J.; [Hohenlohe](https://onlinelibrary.wiley.com/action/doSearch?ContribAuthorStored=Hohenlohe%2C+Paul+A), P.[A.](https://onlinelibrary.wiley.com/action/doSearch?ContribAuthorStored=Hohenlohe%2C+Paul+A); [Bassham](https://onlinelibrary.wiley.com/action/doSearch?ContribAuthorStored=Bassham%2C+Susan), S.; [Amores](https://onlinelibrary.wiley.com/action/doSearch?ContribAuthorStored=Amores%2C+Angel), A.; [Cresko](https://onlinelibrary.wiley.com/action/doSearch?ContribAuthorStored=Cresko%2C+William+A), W.A. Stacks, an analysis tool set for population genomics. *Mol. Ecol.* **2013**, *22(11)*, 3124–3140.

Gosselin, T. grur: an R package tailored for RADseq data imputations. R package version 0.0.10 **2018** https://github.com/thierrygosselin/grur. doi : 10.5281/zenodo.496176

Gosselin, T.; Lamothe, M.; Devloo-Delva, F.; Grewe, P. radiator: RADseq Data Exploration, Manipulation and Visualization using R. R package version 1.1.8 **2020** https://thierrygosselin.github.io/radiator/. doi: 10.5281/zenodo.3687060

Guzinski, J.; Mauger, S.; Cock, J.M.; Valero, M. Characterization of newly developed expressed sequence tag–derived microsatellite markers revealed low genetic diversity within and low connectivity between European *Saccharina latissima* populations. *J. Appl. Phycol.* **2016**, *28*, 3057–3070.

[Guzinski](https://onlinelibrary.wiley.com/action/doSearch?ContribAuthorStored=Guzinski%2C+Jaromir) J.; [Ballenghien](https://onlinelibrary.wiley.com/action/doSearch?ContribAuthorStored=Ballenghien%2C+Marion), M.; [Daguin‐Thiébaut](https://onlinelibrary.wiley.com/action/doSearch?ContribAuthorStored=Daguin-Thi%C3%A9baut%2C+Claire), C.; [Lévêque](https://onlinelibrary.wiley.com/action/doSearch?ContribAuthorStored=L%C3%A9v%C3%AAque%2C+Laurent), L.; [Viard](https://onlinelibrary.wiley.com/action/doSearch?ContribAuthorStored=Viard%2C+Fr%C3%A9d%C3%A9rique), F. Population genomics of the introduced and cultivated Pacific kelp *Undaria pinnatifida*. Marinas—not farms—drive regional connectivity and establishment in natural rocky reefs. *Evol. Appl.* **2018**, *11(9)*, 1582–1597.

Lischer, H.E.; Excoffier, L. PGDSpider: an automated data conversion tool for connecting population genetics and genomics programs. *Bioinformatics* **2012**, *28*, 298–299.

Martin M. Cutadapt removes adapter sequences from high-throughput sequencing reads. *EMBnet. journal.* **2011** *17* *(1)*, 10-12.

Paulino, C.; Neiva, J.; Coelho, N.C.; Aires, T.; Marba, N.; Krause–Jensen, D.; Serrao, E.A. Characterization of 12 polymorphic microsatellite markers in the sugar kelp *Saccharina latissima*. *J. Appl. Phycol.* **2016**, *28*, 3071–3074.
